# Protein-protein binding selectivity and network topology constrain global and local properties of interface binding networks

**DOI:** 10.1038/s41598-017-05686-2

**Published:** 2017-07-17

**Authors:** David O. Holland, Benjamin H. Shapiro, Pei Xue, Margaret E. Johnson

**Affiliations:** 1 Department of Biophysics, Johns Hopkins University, Baltimore, Maryland USA; 2 Department of Biomedical Engineering, Johns Hopkins University, Baltimore, Maryland USA

## Abstract

Protein-protein interactions networks (PPINs) are known to share a highly conserved structure across all organisms. What is poorly understood, however, is the structure of the child interface interaction networks (IINs), which map the binding sites proteins use for each interaction. In this study we analyze four independently constructed IINs from yeast and humans and find a conserved structure of these networks with a unique topology distinct from the parent PPIN. Using an IIN sampling algorithm and a fitness function trained on the manually curated PPINs, we show that IIN topology can be mostly explained as a balance between limits on interface diversity and a need for physico-chemical binding complementarity. This complementarity must be optimized both for functional interactions and against mis-interactions, and this selectivity is encoded in the IIN motifs. To test whether the parent PPIN shapes IINs, we compared optimal IINs in biological PPINs versus random PPINs. We found that the hubs in biological networks allow for selective binding with minimal interfaces, suggesting that binding specificity is an additional pressure for a scale-free-like PPIN. We confirm through phylogenetic analysis that hub interfaces are strongly conserved and rewiring of interactions between proteins involved in endocytosis preserves interface binding selectivity.

## Introduction

Interface interaction networks (IINs), also referred to as structural interaction networks^1, 2^, domain-domain interaction networks^3, 4^, or structurally annotated pathways^5^, are a map of the binding sites proteins use for various interactions. Such a map can be used to model how competition modulates signal transduction^4, 6^; predict the effects of domain mutations on disease^2, 7–9^ and the immune response^10^, predict dosage sensitivity by identifying linear motifs and promiscuous regions^11^, and study the structure and dynamics of multi-protein complexes^12^. For example, Actin can form long fibers because it has a “barbed” end that binds to a “pointed” end of another Actin protein. On a typical protein-protein interaction network (PPIN) map, this interaction would appear as a self-edge, whereas more accurately, they are two distinct binding sites with their own share of possible partners.

We ask four major questions in this work. First, is the structure of IINs conserved across PPINs? Second, does this structure reflect any selective constraints on protein interactions? Third, do the presence of hubs in the PPIN network affect the types of IIN structures possible? And fourth, do hubs in the PPIN provide an advantage (relative to random networks) in producing selective interface interactions with minimal interfaces, suggesting a new benefit for scale-free PPINs? The answer is yes in all cases.

We analyze the structure of four PPINs with IINs defined: two smaller manually curated networks (621 total interactions) and two larger automatically constructed networks (6,893 interactions). Little work has been done on IIN structure, in large part due to the paucity of experimental and crystallography data identifying where proteins bind to one another. The protein data bank^13^ provides the optimal resource for having a computer automatically assign interfaces. However, with limited crystal structures of proteins in complex, homology modeling^5, 14, 15^ is needed to help infer domains and interfaces used for interactions. Interfaces assigned through homology modeling are only putative, however, as this approach is limited in accuracy. The binding sites discovered will depend on the experimental templates used, and even if the sites have similar sequence there is no guarantee of an interaction^15^. Stein *et al*., using known PPIs from six organisms including humans, estimated that less than 30% have a template for comparative modeling^16^. The Interactome3D approach uses several criteria to improve accuracy in predicting binding interfaces, but recovered acceptable models for only ~64% of interactions in their database^5^. Homology modeling will also miss many short linear motif (SLiM)-mediated interactions^17^, both due to their rapid evolution^18^ and low affinity, which has hindered experimental detection^19^. As we see below, limited accuracy in automatically predicted interfaces significantly alters the structure of the IIN, although major features are still visible.

**Figure 1:**
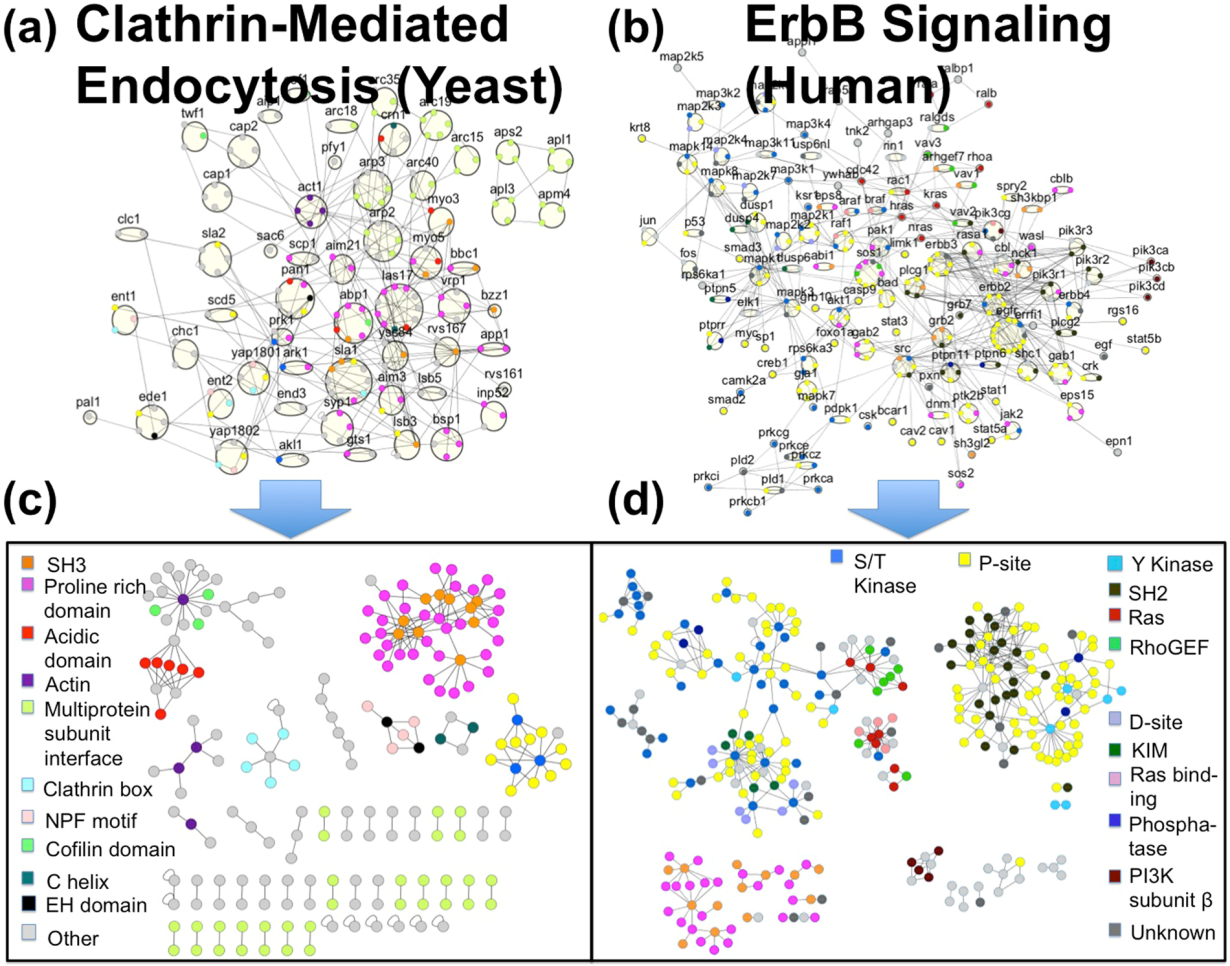
PPINs all contain hub proteins and their IINs have distinctive topologies. We analyze the PPINs of the manually curated yeast endocytosis **(a)** and human ErbB networks **(b)** with all domains and interfaces identified and shown here colored by domain type (Table S2). The resulting interface interaction networks (IINs) in **(c)** and **(d)**, respectively, have highly distinct topologies that reflect the needs of interfaces to achieve strong functional binding and minimize non-functional interactions. Both IINs break into multiple components with a selection of hub interfaces, and they contain an abundance of hub and square motifs with a minimal (or zero) number of triangle motifs.

With manual curation, in contrast, putative interfaces can be refined, corrected, or rejected, and the many protein interactions that lack homology models can be assigned based on detailed biochemical approaches, functional studies, and analysis of disordered regions and SLiMs. So far, two such IINs have been constructed to this gold standard: the clathrin-mediated endocytosis network in yeast^20^, and the ErbB signaling network in humans^4^ (Figure 1). Despite being independently constructed by different research groups, the two share similar features: fragmentation into multiple components, little clustering, and a high frequency of square and hub motifs. With the exception of the presence of hubs, these features differ from their parent PPINs, and thus display a unique topology that we show results due to different selective forces.

For the second question, we propose that one of the selective forces shaping IIN structure is the need to maintain high binding specificity. Due to the chemical nature of binding sites, occasionally nonspecific misinteractions will occur. Avoiding these misinteractions has been demonstrated to be a fundamental force limiting the number of distinct proteins in an organism^21, 22^, protein expression levels^11, 23, 24^, binding strengths^25^, and interface interaction motifs^21, 26^. In regards to IIN motifs, it was found via an amino acid residue optimization model that specific motifs (and not others) and a fragmented IIN structure were needed to optimally design protein interfaces for high specificity^21, 26^. We first compare IIN structures to randomized versions, to demonstrate the biological networks’ clear departure from the statistically most probable IIN structure. We then construct a trainable fitness function to reproduce the observed biological IIN. This fitness function favors network motifs that have been shown to improve the sequence-based binding selectivity of interfaces^26^, and also penalizes high interface diversity. Hence we do not optimize amino-acid sequences, as has been done previously, but rather the network motifs shown to correspond with highly selective sequences^21, 26^. Limiting total interface numbers both lowers the number of possible misinteractions that must be optimized against (order of n^2^)^22^ and mimics the limited size of proteins, which cannot harbor unlimited interfaces. Because the search space for possible IINs of a given PPIN is enormous (quantified below), we used a Monte Carlo sampling algorithm combined with a fitness function (Methods) to find the optimal IIN at various parameterizations, similar to previous work optimizing spatial networks^27^.

Because the automatically constructed^1, 2^ IINs contained systematic errors, largely due to missing SLiMs as binding partners and incorrect replacements, we restricted our training and sampling procedure to the two manually curated networks. However, this outcome highlighted a powerful advantage of visualizing the IINs: the network motifs can be used to identify erroneous domain-domain interaction predictions. Disagreements over the evolution of proteins and their networks can often be attributed to variability and poor overlap in PPIN datasets^28^. Boosting domain assignment accuracy by identifying errors in automatically constructed networks using network motifs, as we demonstrate here, improves these crucial resources for understanding protein function and evolution.

To address the third question and learn how the presence of protein hubs affects the IIN sampling space, we combined both analytical and computational sampling approaches to characterize the structure of IINs as a function of varying PPIN structure. PPINs feature a degree distribution that is approximately power-law or “scale-free”, meaning (loosely speaking) that a few proteins act as hubs, while the majority of proteins are specialized to only a few interaction partners^29^. This same basic structure describes airport networks, and is the optimal structure for maximizing transport with minimal costs^30^. By considering the possibility of a random PPIN, we can then compare whether this alternative structure is different and possibly worse than a scale-free PPIN in terms of IINs possible. For example, a well-known advantage of scale-free PPINs relative to random networks is their ability to maintain connectivity under attack^31^. Because IINs have not been studied in the context of their parent PPIN, we first establish how the whole domain of possible IINs varies with PPIN structure, showing that hubs do alter the space of IINs in specific ways.

For our fourth question, we sought to test whether the real PPINs were any better for developing selective binding than the random PPINs. We applied our data-trained fitness function at its optimal parameters to sample IINs for scale-free versus random PPINs of the same size. Random PPINs proved more difficult to optimize, requiring the evolution of significantly more interfaces (penalized in our fitness function) in order to achieve the same level of binding complementarity encoded in the IIN motifs. This runs counter to the parsimonious use of domains across species, where new domain combinations rather than new domains drive functional divergence^32^. Ultimately our result suggests an additional pressure for a scale-free-like PPIN. It is a cheaper (fewer interfaces) design for maintaining a multitude of selective binding interactions.

Our model emphasizes that selectivity in interface binding is critically conserved across IINs, and that hubs in the PPIN provide an advantage in this regard, largely because they may contain hub interfaces. As a final analysis we use phylogenetic analysis to test whether interface binding selectivity is conserved as protein-protein interactions are rewired throughout evolution^33^. We use this analysis to test whether, despite this rewiring, hub interfaces are nonetheless conserved, providing a new physico-chemical argument supporting the conservation of hub proteins.

## Results

### IINs for the biological PPINs have highly specialized features sensitive to rewiring

To determine if IIN structure is conserved across PPINs, we first characterize the manually curated PPINs from yeast and humans shown in Fig. 1a,b (Table S2), which involve different protein sets but both exhibit scale-free-like topologies. Analysis of both their IINs (Fig. 1c,d, Table S2) demonstrates that they both share highly similar features to one another and are topologically unique. They have fragmented structure, almost no triangle motifs (low C_global_), a higher fraction of hub versus chain motifs, and a significant fraction of square motifs (Table 1). In contrast, expected values for these features, calculated by randomly rewiring the interface interactions while keeping the PPIN structure intact, have no similarities (Table 1). Rewired IINs organized into a giant component with many chains, increased triangles (higher clustering coefficient C_global_), and minimal squares (Table 1, Fig. S1, Table S6). The lack of hub interfaces in these rewired IINs is reflected by the low preferential attachment exponent (P.A.E.), which varies from 0 for random networks to ~1 for scale-free networks (Methods).

**Table 1:** Comparison of properties of the IINs from two manually curated PPINs and two automatically constructed IINs.

	Yeast CME IIN	Human ErbB IIN	Human SIN	Yeast SIN^a^
Proteins	56	127	3626	167
PPIN Edges	186	268	6585	308
Interfaces	195 [200]	297 [411]	5494	308
IIN Edges	206 [207^b^]	415 [420^b^]	11,466	539
Self Loops	10	2	3414	0
IIN PAE	0.8 [0.09 ± 0.09]	0.7 [0.24 ± 0.07]	1	1
LC^c^ (PPIN)	92%	100%	43%	36%
LC^c^ (IIN)	23% [82 ± 4.0%]	35% [96 ± 2%]	33%	35%
C Global	0 [0.016 ± 0.01]	0.002 [0.01 ± 0.005]	0.17	0.21
Tetramers	2,743 [819 ± 92]	10,856 [4,312 ± 280]	2.5 × 10^6^	16,530
Squares	0.061 [0.002 ± 0.002]	0.066 [0.005 ± 0.001]	0.0210	0.0557
Hubs	0.56 [0.26 ± 0.020]	0.58 [0.27 ± 0.01]	0.461	0.339
Chains	0.37 [0.73 ± 0.02]	0.36 [0.72 ± 0.01]	0.374	0.455

Bracketed values are expected values for IIN properties with standard deviations, see Supplemental Text S2 and S4 for further details on calculations.

^a^Only the cytoplasmic proteins used in (Deeds *et al*., 2012).

^b^Edges numbers were capped when sampling to prevent continuous growth.

^c^Percent of nodes in largest component of network.

The structure of the two automatically constructed IINs^1, 2^ was in some ways similar to the manually curated IINs, but they are closer on the spectrum towards a randomly rewired network. Similar to the manually curated networks, they have a large PAE, indicating hub interfaces in the network, and a similar fraction of square motifs (Table 1). They also have correspondingly more hub motifs in the network than would be observed in a random network. A significant difference is the degree of fragmentation. The manually curated networks are nearly fully connected at the PPIN level, and yet the IINs contain a largest connected component of only 23–35% of nodes. In contrast, Human SIN^2^ is already fragmented at the PPIN level (43% of nodes in the largest component), and the IIN fragmentation is therefore more strongly driven by the PPIN fragmentation. The Yeast SIN^1^ is even more dramatic. The reason for the higher connectivity in these IINs is the larger ratio of chain to hub motifs (Table 1), as chain motifs prevent fragmentation into many distinct modules (Fig. S2). The number of triangle motifs, which is directly quantified by the clustering coefficient C_global_, is also significantly higher in these networks than in the manually curated networks (Table 1). Does the increased randomness of these IIN connections occur due to mis-identification of interaction interfaces? By following up on this implication by investigating the many unexpected triangles in the automatically curated IINs, we found this was true (Fig. S2 and Supplementary Text S5).

We found mis-assignments of interface interactions can be largely attributed to a lack of linear motifs included as potential binding partners, and a permissive decision-making algorithm. Application of the INstruct website^34^ to predicting CME protein interface interactions produces only 44 interactions (versus 206 for the manually curated network of Fig. 1a ^20^). Of these 44 predicted interactions, only 1 defines the correct domains (Fig. S2). This method predicts a disproportionate abundance of homo-dimers. Many interactions are predicted to be SH3-SH3 interactions (including in the Human SIN^2^ (Fig. S2)), but even in the crystal structures, SH3 domains form homo-dimers only in special cases when mediated by a ligand (such as a PRD)^35^. We also note that some structured domains (such as kinase domains) must be recognized as containing multiple protein binding interfaces. Many kinase domains, for example, form dimers through distinct interfaces and can still perform catalysis^36^.

### Network motifs in the IINs indicate suppression of nonfunctional interactions

**Figure 2:**
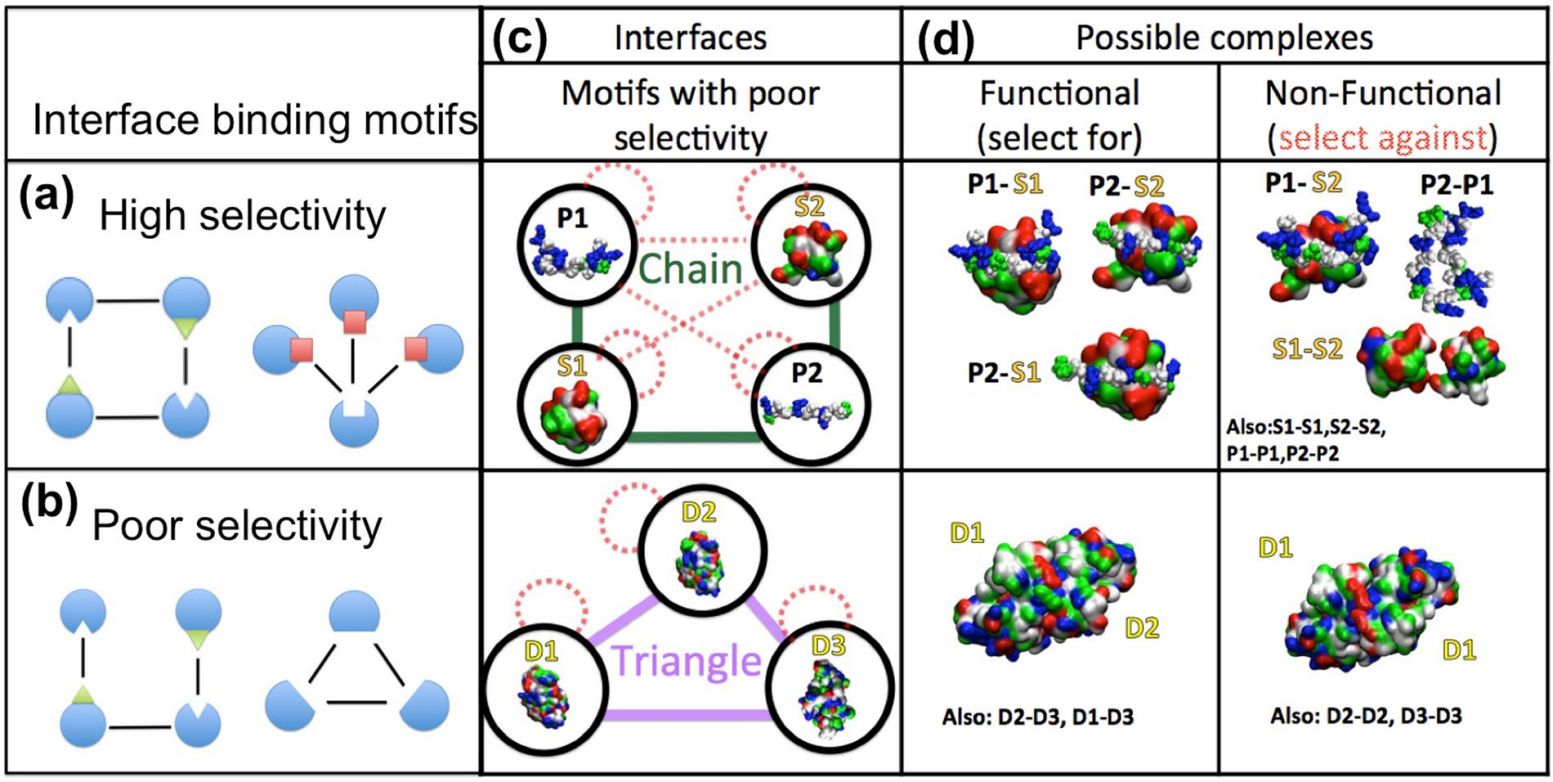
Motifs uncommon in the biological IINs due to poor interface binding selectivity. (**a**) IIN motifs that confer high selectivity. Binding partners may achieve structural and chemical complementarity with few constraints. (**b**) IIN motifs with poor selectivity. (**c**,**d**) For the motifs with poor selectivity, (chain and triangle motifs) functional interactions are indicated by solid lines and interfaces should be optimized to stably form these complexes. However, the possible non-functional interactions between the interfaces (red dashed lines) are difficult to simultaneously optimize for much weaker binding because they share structural and chemical complementarity to the functional complexes. For the chain motif, illustrated with SH3 and PRD domains, the top interaction (S2-P1) is hard to prevent. For the triangle motif, illustrated with kinase domain interfaces that form side-by-side dimers (B-raf, Raf-1^36^, Ksr1) the homo-dimer binding interaction is hard to prevent. In fact, these kinase interfaces really do form both hetero- and homo-dimers, so the biological system has no challenge for optimizing selectivity. Binding surfaces are colored by residue as non-polar (white), polar (green), acidic (red), and basic (blue). Example structures from 2RPN, 2LCS, 1UWH, 3OMV.pdb. Truly non-functional interactions (i.e. PRD-PRD) are just illustrations.

For our second question, we connect the special conserved structure of the biological IINs (Fig. 1 and Table 1) to constraints on binding selectivity. In previous work, using Monte Carlo based optimization of amino acid sequences in small networks, it was shown that when interface interactions were mediated by hub or pair motifs, and not chain motifs, the binding selectivity (measured via the energy of binding interactions) of the interfaces was significantly higher^21^. Thus the level of achievable binding complementarity and selectivity is encoded in these basic motifs, which include hubs, pairs, and chains. Subsequently, it was shown that IINs were also more selective if they were highly fragmented into modules^26^. In both cases this is because it is easier to optimize the interface sequences for both strong specific interactions, and against non-functional mis-interactions. All of these trends are clearly present in the biological IINs, and not the random IINs (Table 1). In Fig. 2 we further illustrate how, for the same reason, square motifs are beneficial to selectivity, and triangle motifs are detrimental. While it is perfectly possible to design interfaces that will bind strongly in any motif configurations, the real challenge is to simultaneously suppress the nonfunctional interactions possible for those motifs. For the chain motif, the challenge is preventing the interaction between the two ends of the chains. For the triangle motif, in order for all three distinct domains to attract one another, they must all be similar to one another. If an interface binds a very similar interface to itself, it will likely also bind to itself. Thus, triangle motifs are only consistent with high-selectivity optimization if their interfaces are also self-binding. We found that for the one triangle present in the ErbB IIN, this was indeed the case. Two kinase domains form not only a heterodimer with a shared target, they also both form homo-dimers^36^, and hence we added these previously undefined self-interactions to the network.

### The space of possible interface networks for a PPIN is enormous and varies with protein degrees

**Figure 3:**
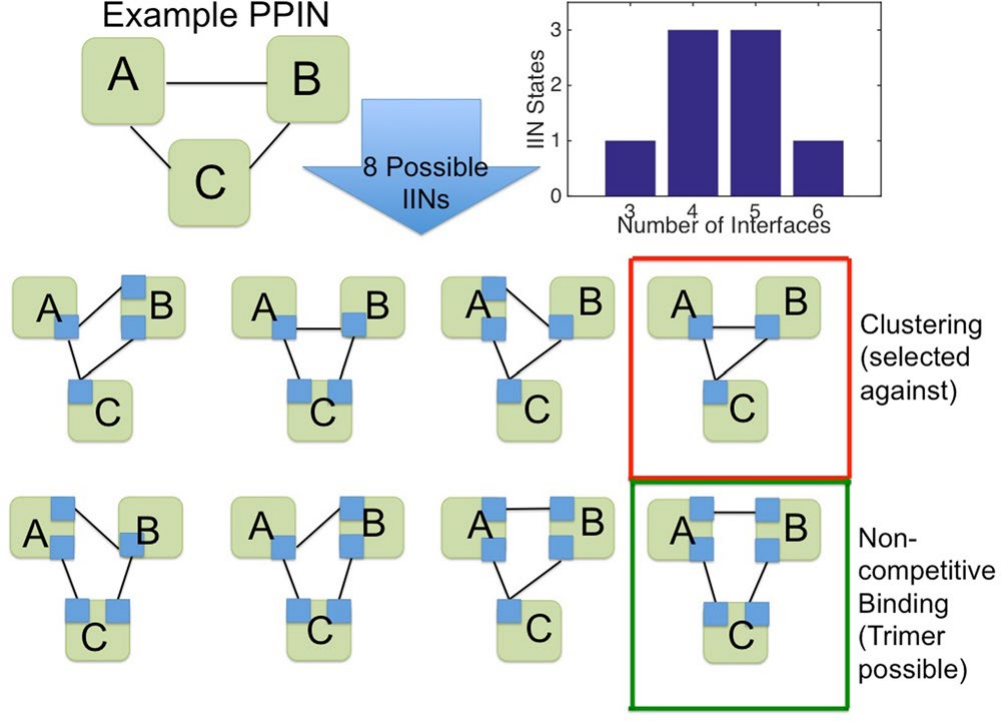
Each PPIN has many possible IINs, and only some are good for promoting selectivity. For the simple PPIN with three interacting proteins, there are 8 possible IINs with either 3, 4, 5 or 6 interfaces (blue squares). Because each IIN has different motifs present, only a subset will be favored in biological networks. The top row contains IINs with chain motifs or a triangle motif (red box), which are bad for promoting selectivity and less common in biological IINs. The bottom row contains favorable motifs, and in the green box is the only IIN that allows a true protein trimer to form. IINs with 4 or 5 interfaces are most common, as counted in the histogram. The same trend holds for much larger PPINs, with the sparse and dense IINs becoming increasingly rare, and hub interfaces less common.

Our third question considers how the PPIN structure might constrain the IINs accessible. While a PPIN and its interface interaction network (IIN) must evolve together, it is not obvious how one constrains the other, given that a protein can use one or many interfaces for its various partners. To illustrate properties of IINs constrained to a PPIN, in Fig. 3 we enumerate the 8 possible IINs for the simple PPIN of three proteins binding. The total number of possible interface networks is determined by the number of interactions (degree, *k*) per protein and quantified through the Bell number B_k_. Bell numbers grow rapidly and hence high-degree hub nodes can dramatically increase the number of possible IINs, meaning a scale-free PPIN will have significantly more IINs possible than a random PPIN because of its hubs. We calculate 10^166^ IINs for the clathrin-mediated endocytosis (CME) PPIN in Fig. 1a, and 10^143^ for a similarly sized random PPIN (more than the number of atoms in the universe!) (Table S1, Supplementary Text S4). Both types of PPINs produce IINs with an expected degree distribution that is random, not scale-free. This is because configurations that create hub interfaces, which are necessary to produce a scale-free IIN, are rare. However, hub proteins do cause several subtle shifts in the properties of the IINs possible, including slightly fewer expected interfaces, more 4-node motifs (tetramers) and more hub interfaces (Table S1, Supplementary Text S4). Since these are the features important in the biological IINs, this is an indication that the hub proteins found in scale-free PPINs may promote more selective IINs.

### Strong motif biases are needed to reproduce biological IINs

To answer question four, and address whether the PPIN structure influences the ability to produce biologically optimal IIN structures, we first needed to be able to sample biologically realistic IINs given a PPIN. To do so we created a fitness function and trained it to reproduce the networks of Fig. 1. Due to the inaccuracies of the automatically constructed IINs (Table 1), we did not include them to avoid training the fitness functions towards erroneous network structures. The fitness function is biologically motivated to penalize features that promote mis-interactions, to not penalize features that promote strong interactions, and to capture physical size constraints of proteins. We therefore included a bias against triangle subgraphs without self loops (parameterized by β) and chain subgraphs (parameterized by κ), which are difficult to optimize for structural and chemical complementarity as explained above (Fig. 2). These two separate terms resolved a problem we found with our previous fitness function^26^: this earlier approach did penalize chain subgraphs, but it also ended up penalizing biologically realistic square subgraphs. Our current fitness function does not penalize squares. We introduced a third parameter, μ, to penalize having large numbers of interfaces in the network, both because this increased diversity leads to more possible misinteractions^22^ and because proteins have limited volume for extra interfaces. Finally, in the biological IINs, protein pairs can interact through multiple domains, resulting in a significant increase in edges from the PPIN to the IIN (Table 1). Our fourth and final term thus allowed new duplicate edges in the IIN but limited their growth by a parameter ω. All four parameters are dimensionless and weight topological properties of the network (see Methods for details and illustrations).

**Figure 4:**
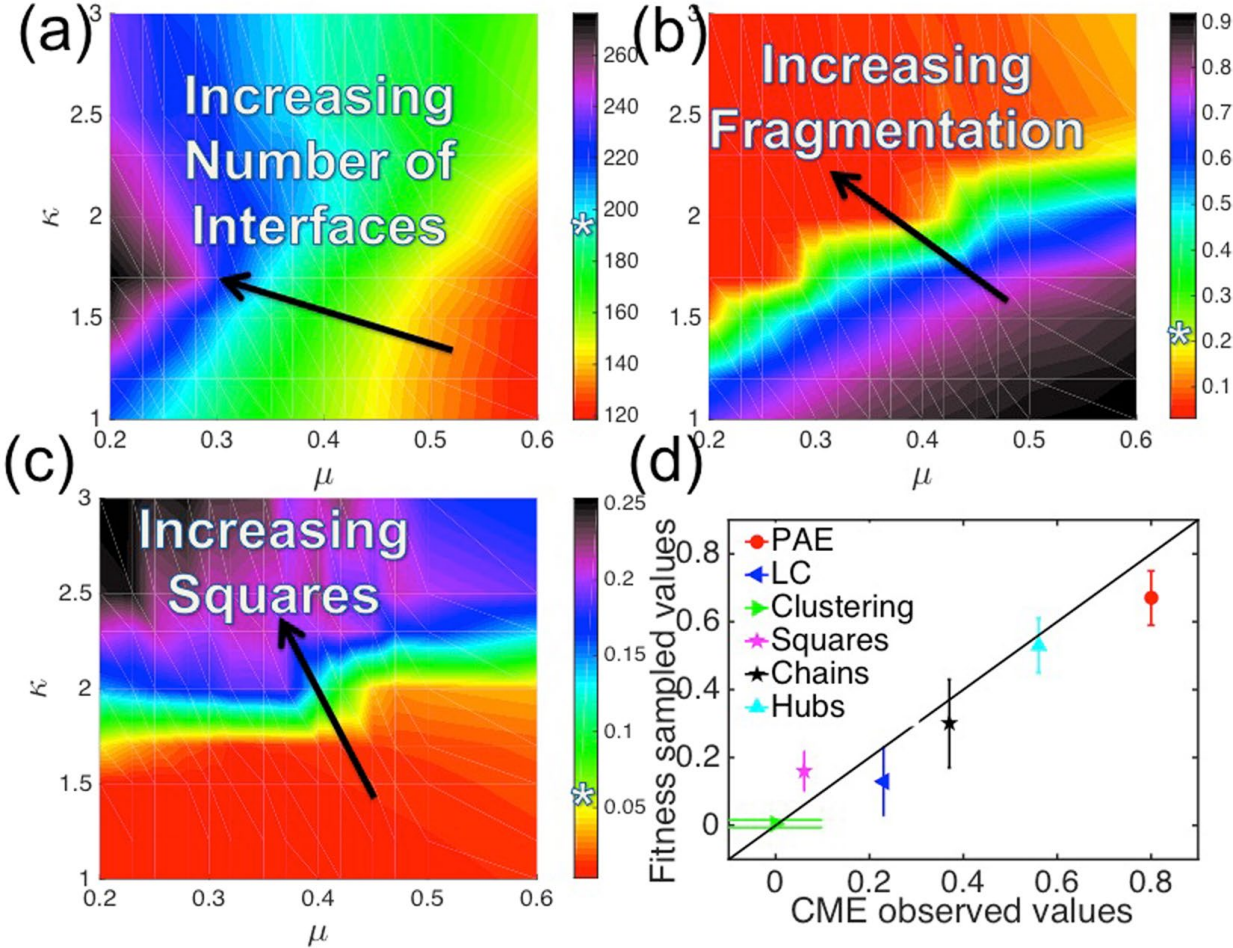
Learning how to select biologically realistic IINs for a PPIN using a parameterized fitness function. Because biological IINs are so distinct from a randomly generated IIN, we needed a four parameter fitness function to bias the sampling towards the correct: (**a**) number of interfaces (**b**) size of the largest module/fragment (**c**) Frequency of square motifs in the IINs, as well as other properties (Fig. S3). The results were most sensitive to variation in the parameters κ and μ (on the axes) that regulated the square-to-chain ratios and number of interfaces, respectively, in the fitness function. White stars on color bars indicate observed values of the CME PPIN (Fig. 1a). (**d**) By training the fitness function, we achieved very good agreement between the properties of the sampled IINs and observed CME IIN with optimal fitness parameters κ = 2, μ = 0.42, β = 4 and ω = 0.1. Comparable parameters applied to the ErbB PPIN (κ = 2.3, μ = 0.45, β = 4) except we lowered ω to 0.02 to account for the much greater frequency of edge duplication.

We had to optimize the four parameters of our fitness function to locate the biological IINs out of the enormous space of possible IINs (e.g. 10^166^), where all parameters were greater than or equal to zero, and setting a parameter to zero effectively turned off that fitness pressure on the IINs (Fig. S3). All four parameters were needed. We found that the key to generating realistic IIN features required a balance of creating new fragmented modules without introducing too many interfaces. To do so required re-using interfaces that would generate either isolated star hubs (e.g. turquoise nodes in Fig. 1c) or hubs connected in square clusters (e.g. orange and pink nodes in Fig. 1c,d). In Fig. 4 we show how the most important parameters for simultaneously capturing these dominant features of the IINs were κ and μ. The parameter κ penalizes chains and μ penalizes the creation of new interfaces, and together they exhibit the most sensitive control over the IIN structure (Methods). Star hubs, like squares, result from pressure to avoid chains and hence are also positively selected for with increasing κ (Fig. S3). Our trained fitness function samples IINs with very close agreement to the observed CME network (Fig. 4d, Fig. S3) with parameters κ = 2, μ = 0.42, β = 4 and ω = 0.1. Comparable parameters applied to the ErbB PPIN (κ = 2.3, μ = 0.45, β = 4) except we lowered ω to 0.02 to account for the much greater frequency of edge duplication. In the discussion we consider ways to further improve the agreement.

### PPINs need hubs to minimize new domain interfaces

**Figure 5:**
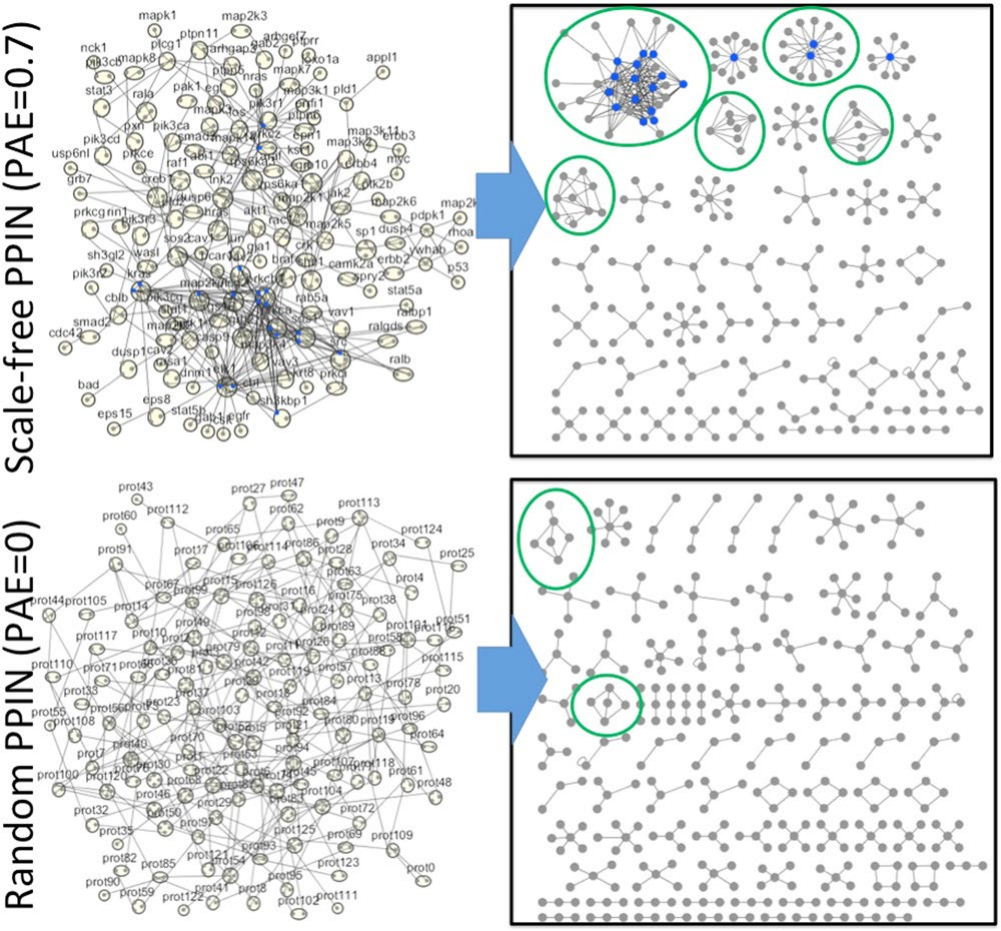
Scale-free PPINs produce fitter IINs than random PPINs. We performed fitness sampling for selective IINs on the ErbB scale-free like PPIN (top) and a random network with the same number of proteins and PPIs (bottom). For the scale-free like PPIN (top) fewer interfaces (n = 290) were needed to produce selective motifs, including 2000 squares (in green circled modules). Without hub proteins, the random PPIN (bottom) produced only 12 squares, and introduced many additional interfaces (n = 356) in order to maintain selective motifs. The same trends held with the CME PPIN (Fig. S1). IINs discovered with random PPINs were also less fit than those found with scale-free PPINs (see Table S7). Nodes with >9 partners are shown in blue.

We used our trained fitness function to sample IINs for a variety of PPIN topologies and sizes and therefore answer our fourth question. We compared the CME and ErbB PPINs with PPINs of the same size but a random degree distribution, and performed the same experiment for new PPINs both more and less densely connected than these (Fig. S4). Regardless of the size of the PPINs, we found that because random PPINs lack hub proteins, they cannot produce selective domain modules without significant addition of new interfaces (Fig. 5). Thus random PPINs do have a disadvantage, as evolving more interfaces is a more costly way of mediating protein-protein interactions than re-using domains already optimized for selectivity.

The main advantage of hub proteins in a PPIN is that they are capable of more highly connected hub interfaces in the IIN. Although hub interfaces are still possible for a random PPIN of sufficient density (Figs S4 and S5A), the reduced size and frequency of these hubs limits how many square motifs can form (Fig. S5B). Square cluster components are a prominent feature of the biological IINs and they are critical for maintaining selectivity with a minimum number of interfaces. Without access to these motifs, random PPINs require more interface splitting to instead produce selective star hubs. These results were robust to changes in the fitness function that allowed larger fluctuations in interfaces per protein (Supplementary Text S1, Fig. S1). Ultimately, our results suggest that a scale-free-like PPIN is beneficial to evolving specificity in interface binding interactions.

### Network rewiring maintains selectivity

**Figure 6:**
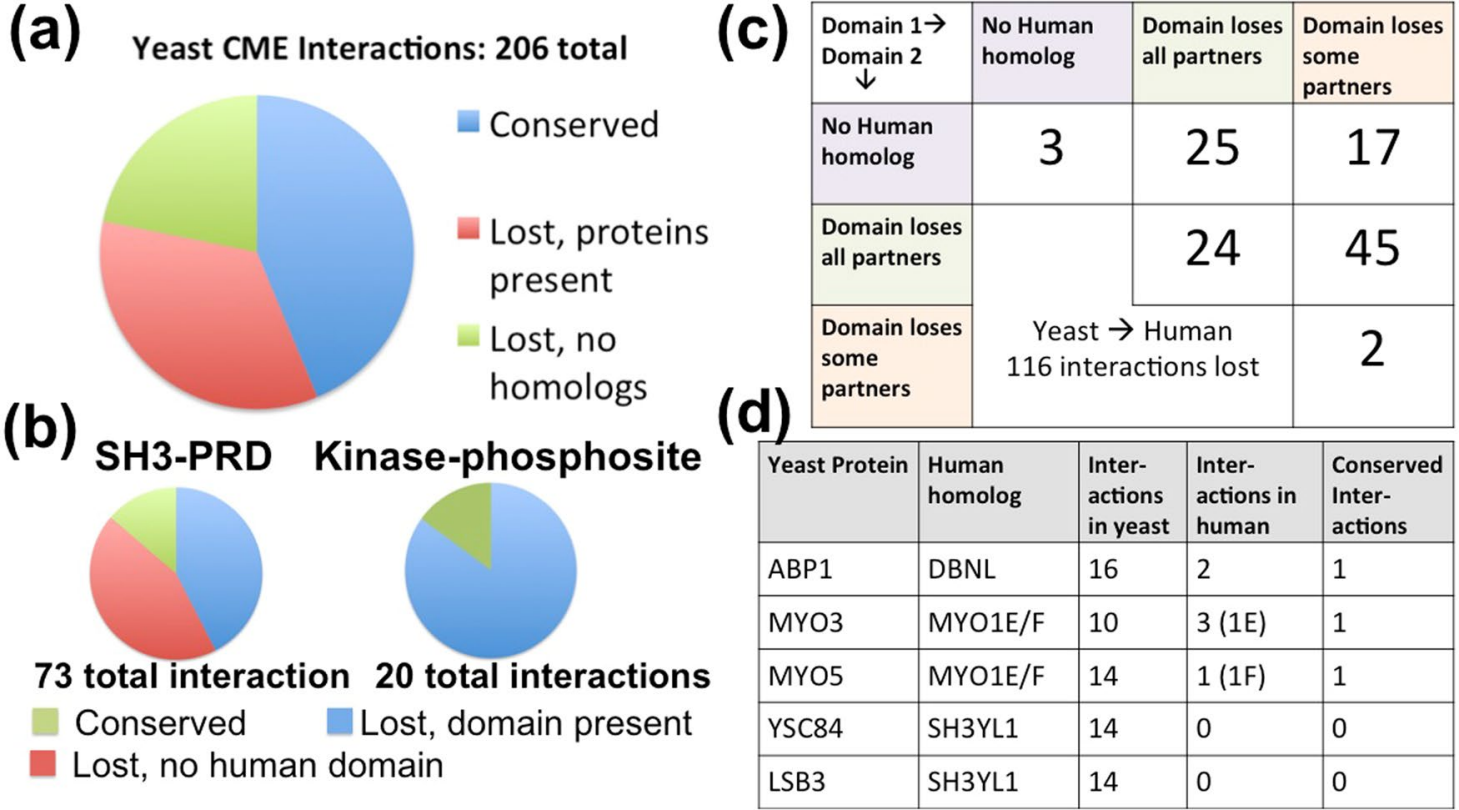
Network rewiring between yeast and human CME networks is correlated and controlled by specific domains. (**a**) Comparison of the CME interactome of 56 yeast proteins with that of their 64 human homologs reveals the majority of interactions are either conserved or lost from yeast to humans due to a missing homolog in the human network. Analysis of changes from the human to the yeast interactome in Fig. S6. (**b**) Of the interactions lost from yeast to human, they were highly correlated, with most being lost due to a full protein homolog being lost, or a domain losing all binding partners. (**c**) Most lost interactions involved SH3 and proline rich domain (PRD) interactions, or kinase-phosphosite interactions, highlighting the fluidity of linear motif driven interactions. (**d**) Some yeast proteins conserved almost no interactions with human counterparts, and these proteins contain SH3 domains.

Our results imply that selectivity in interface interactions is highly conserved across various protein networks. Therefore, if we compare IINs across evolution, we should find that rewiring of interactions between species is not random (as they are treated in growth models) but correlated and constrained to maintain this selectivity. Orthologous proteins with similar domain sets may change protein interactions but should preserve domain partners, as has been experimentally observed in SH3 domain interactions between worms and yeast^37^. By comparing the yeast CME PPIN with a human CME PPIN constructed (Methods) from 64 proteins with recognized functional homology^38^ (Table S4), we find that rewiring events are highly correlated and attributable to specific binding domains (Fig. 6). From yeast to humans, about half of the interactions are conserved. Of those that are lost, 39% are due to lack of a homologous protein, and 98% of the remainder involved at least one domain that retained no interaction partners (Fig. 6C, Table S5). A major source of divergence was domains targeting the linear motif proline rich domains (PRDs) and phospho-sites (Fig. 6B). SH3-PRD interactions accounted for over half the losses from yeast to humans. The divergence of these interactions can be attributed to the biological distinctions between yeast and metazoan CME: in yeast the actin cytoskeleton is required to deform the stiffer cell membrane and the SH3 containing proteins link the cytoskeleton to the clathrin-coated vesicle^38^. New interactions gained within the human PPIN were concentrated in a few proteins, most significantly in the AP-2 complex (Fig. S6, Table S5). The source of these new interactions is an added appendage domain to the human AP-2 complex that interacts with a range of diverse binding partners^39^. Without this hub domain, the yeast AP-2 complex evolved with few binding partners, accounting for the minimal interaction conservation between the homologs.

### Hub interfaces in the CME and ErbB networks are strongly conserved

Our results also emphasize the importance of hub interfaces to avoid the need for new domain innovation. We thus predict hub interfaces should be preferentially conserved throughout evolution. With all the domain information available for the two manually curated networks (Fig. 1), we can isolate the contribution of hub interfaces to hub protein evolution. Hub proteins may evolve more slowly^40^, and one (among other^41^) rationale is that it is harder to change with so many binding partners. However, a conflicting observation is that hub proteins also have more disordered regions^42^, which evolve more rapidly^43^. Furthermore, a distinction between evolutionary rates of different hub types (date vs party hubs) may actually be attributable to expression levels^44, 45^, which, along with number of translational events^46^ are the strongest predictors of evolutionary rates^47^. Our analysis (Methods and Table S3) of residue conservation demonstrates that hub interfaces (defined in two independent ways) are significantly more likely to be conserved than other binding interfaces, with almost 90% being strongly conserved, compared with 70% of non-hub interfaces (Table S8). Because we evaluate conservation on both hub and non-hub interfaces of the very same proteins, the effects of protein expression level variation towards conservation are automatically accounted for. Whether a protein has high or low expression, its hub interfaces are more strongly conserved than its non-hub interfaces. It is the interfaces that bind to the hub interfaces that are more likely to have weaker conservation (Table S8), hence facilitating the growth and rewiring to hub interfaces. This analysis thus directly explains how many hub proteins can participate in more rewiring events^33^, but still evolve slowly: the partners are the ones evolving to achieve binding.

### Hub proteins and disordered regions

Lastly, to better characterize the hub proteins in our network, some of which do not contain hub interfaces, we assessed the role of disordered regions in hub proteins for mediating interactions. We found that hub proteins with few interfaces, and thus more highly connected hub interfaces, were less likely to use disordered regions to mediate interactions (Fig. S7). This is expected because hub interfaces are highly conserved (Table S8), and are thus unlikely to be disordered regions such as PRDs, which have low conservation (Table S8). Examples from our two networks are the kinase PRK1, which uses its kinase domain for ~83% of interactions, and the ErbB proteins MAPK1 and PIK3R1. Conversely, hub proteins with many interfaces, and thus without hub interfaces, used disordered regions to mediate a significantly larger fraction of their interactions. Examples include the hubs LAS17 and ABP1, which use disordered interfaces for 78% and 46% of interactions, respectively. But many hub proteins fall in between, existing on a stratum between having several unstructured binding regions and having a few highly connected structured binding domains (Fig. S2).

## Discussion

PPINs feature a scale-free-like topology. Much like airport networks, a few proteins act as hubs, while the majority of proteins are specialized to only a few interaction partners. Stochastic growth models^48–50^ provide a simple explanation for how protein networks acquire a scale-free topology. Hubs are generated via protein genes duplicating and diverging^48, 51^, where at least one of the duplicated proteins retains an original interaction as they sub-functionalize^52, 53^. While gene duplication and divergence is undoubtedly a source of evolutionary changes to protein interactions, the network growth models of duplication and divergence have an unrealistic portrayal of rewiring, usually performing only one rewiring per duplication event, and without incorporating any physico-chemical or evolutionary basis for the rewiring. Rewiring happens on a much faster evolutionary timescale than gene duplication: the human interactome has been estimated to rewire 1000 times per million years^33, 51^, whereas gene duplication is estimated to occur at a rate of 2 to 30 events per million years^54, 55^ (assuming 20,000 genes), with the majority of these duplications being deleted by natural selection^56^. Orthologous proteins between species are often highly rewired, as a recent study comparing the yeast and worm SH3 interactome found^37^. Additionally, growth models ignore homo-dimers despite their prevalence^57^ and influence on evolving new interactions^58^.

Biological rewiring is capable of abolishing the majority of interactions from one species to another^59^, and creating and destroying transcription factor^60^ and protein hubs such as AP-2^61^ between species^38^. If the rewiring were random, it would destroy any scale-free structure created by gene duplication. Yet scale-free topology is conserved, and this suggests rewiring is not random and hubs are preferentially conserved^51^. A scale-free topology is known to provide benefits relative to a random network in that it fortifies communication across networks by centralizing connections into hubs^31^. We propose that our results provide another advantage of hubs in PPINs: they improve binding selectivity and avoidance of misinteractions. This selection pressure is of molecular origin and reflects directly on the primary physico-chemical requirements of proteins to fold into stable structures and bind to other molecules. Hub proteins allow the creation of hub interfaces, which facilitates chemical and structural complementarity and selectivity with the fewest number of interfaces needed.

We note that the actual IINs were not the most optimal solutions in any fitness landscape. Raising the temperature allowed us to sample more randomized versions of the optimal solutions, but the real IINs departed from the optimum in specific, rather than random ways, suggesting additional selective pressure acting on the network structure. Firstly, the real IINs had a smaller number of isolated modules. Each large module corresponds to a particular binding mode; e.g. SH3 to PRD or Ras to GEF interactions. Cells have a limited number of domain/interface types to work with, but our model only limited total interface numbers and not types, as we did not assign types to interface nodes. However, one way we could capture this selection pressure on interface types is by applying selection pressure in our sampling against the total number of modules. The same motif structure in fewer modules would better match the observed biological IIN structure and also mimic the limited number of domain types used by proteins. Secondly, our fitness function applied selection pressure against motifs that were sub-optimal in terms of binding selectivity, but in some cases, these interactions may be optimal in terms of function without truly sacrificing selectivity. How? They can be essentially turned on or off by regulation such as phosphorylation or allostery. This is especially true of “bridge” interfaces that connect otherwise separate modules. The ARC40 subunit of the ARP2/3 complex acts as a bridge node in the CME IIN that can be inhibited from binding actin^62^. However, it is difficult to select for functional constraints without knowing the true function of every protein in the network, and even then function is not a generic constraint; it would have to be selected for in a targeted way. It is noteworthy however that we are able to reproduce key features of the IINs without the need for incorporating protein function.

Finally, it is estimated that at least 40% of proteins bind to themselves, and the majority of these interactions involve a homo-dimer using the same interface^57^. In networks, however, these interactions produce self-loops that are often ignored when calculating network properties and simulating network growth, despite providing a justification for frequent paralog interactions in growth models^58^. They are ignored because having another unique edge type increases the combinatorial complexity of network structures, but we found here that they are critical in correctly capturing motif selectivities. This is best illustrated by the triangle motif in Fig. 2 that switches from low to high specificity with the introduction of multiple self-interactions. The optimal selectivity for a self-binding interface is as an isolated node, or as part of a pair of hetero-dimer forming homo-dimer interfaces, as is clearly evident in the CME IIN (Fig. 1a). Self-binding nodes are least selective as hub interfaces because suppressing non-functional interactions grows more difficult with more partners that are not self-binding. These distinctive motif preferences for self-binding interfaces present another important consideration for curating domain assignments in PPINs, in this case suggesting both potential mis-assignments and missing assignments.

## Methods and Models

### Fitness function to sample IINs on a PPIN

**Figure 7:**
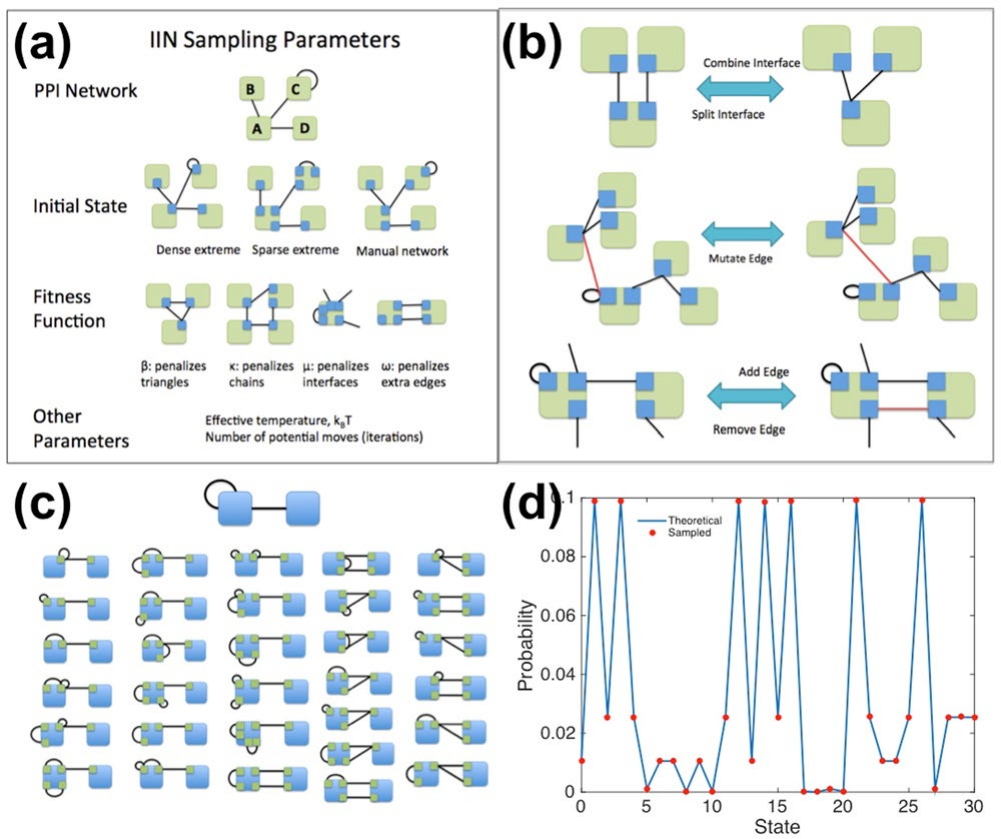
Interface networks for a given protein network can be sampled via Monte Carlo methods with or without bias. (**a**) Inputs and parameters for our stochastic IIN sampling model for a given PPIN that is not altered. (**b**) Monte Carlo reversible move sets (5 moves possible) to transition between IIN structures. (**c**) A two protein network with 2 PPIs can be enumerated into 31 distinct IINs when one extra edge is allowed. Moves between states were enumerated as a Markov chain to determine the factors necessary for detailed balance. (**d**) Proof of detailed balance in the toy model (C). The probability of being in a given state is proportional to its propensity *e* ^*−f/kBT*^, where “f” is the assigned fitness penalty (low “f” = more fit) and k_B_T is set to 2. The blue line is the theoretical stationary distribution based on propensities, and the red circles are the MC sampled results.

Given a fixed PPIN, we used Monte Carlo sampling in the space of IIN structures with networks structures accepted or rejected via the Boltzmann weight ${e}^{-({f}_{new}-{f}_{old})/{k}_{B}T}$. The four parameter (ω, β, κ, μ) fitness function given by

1 $$f={e}^{\omega ({M}_{IIN}-{M}_{PPI})}+{\sum }_{i,({k}_{i} > 1)}^{{N}_{\mathrm{int}}}({e}^{\beta {C}_{i,3}}+{e}^{\kappa (1-{C}_{i,4s})}-2)+{\sum }_{p}^{{N}_{pro}}{e}^{\mu {N}_{\mathrm{int},p}}$$

controlled the numbers of interfaces *N*_*int*_ and edges *M*_IIN_ in the IINs, as well as the triangle motifs and square-to-chain motif ratio via the local clustering and grid coefficients^63^, *C*_i,3_ and *C*_i,4s_.

2 $${C}_{i,3}=\frac{2{N}_{triangle,i}}{{k}_{i}({k}_{i}-1)}$$

3 $${C}_{i,4s}=\frac{1+{N}_{square,i}}{1{+}^{{k}_{i}^{2nd}{k}_{i}({k}_{i-1})}/2}$$

where *k*_*i*_ is the degree of node “i”, *k*_i_^2*nd*^ is the number of nodes two steps away from “i”, and *N*_*triangle,i*_ and *N*_*square,i*_ are respectively the number of triangles and squares which pass through “i”. A dummy square (+ 1 term in numerator and denominator) in the grid coefficient is used to penalize having a high number of chains even when *N*_*square,i*_ equaled zero. Triangles on which at least two of the nodes had self-edges were ignored, since this is not a constraint against high specificity. The fitness function penalizes having a high clustering coefficient (many triangles), a low grid coefficient (many chains), a high number of interfaces, and it penalizes duplicating too many edges (Fig. 7).

### Monte Carlo sampling of networks

We first initialized the IIN structure to either the dense extreme (one interface per protein), the sparse extreme (new interface per each edge), or the known IIN structure. Moves (illustrated in Fig. 7) were accepted or rejected based on the Boltzmann criteria, where we were careful to ensure detailed balance given the different probabilities of generating forwards and reverse moves (*p* ^*gen*^) via the acceptance probability:

4 $${p}_{II{N}_{old}\to II{N}_{new}}^{accept}=\,\min (1,\frac{{p}_{reverse}^{gen}}{{p}_{forward}^{gen}}{e}^{-\frac{({f}_{new}-{f}_{old})}{{k}_{B}T}})$$

where *f* is the fitness of the IIN defined in Eq. 1, and *k*_B_ *T* is the effective temperature. We verified our implementation for a small test network in Fig. 7. The entire space of possible IINs could be sampled by setting k_B_T = ∞. For the fitness sampled IINs, we found a range of k_B_T = 0.1–1 to be optimal. Modified versions of sampling to test the robustness of our network properties are described in Supplementary Text S1.

Simulations were allowed to equilibrate for the first 1/5 of the total number of iterations, (usually ~1 million iterations) after which the statistics of each network sampled was recorded so as to record average statistics favored by the fitness function. The best-fit (lowest fitness penalty) network discovered was also recorded.

### Optimizing fitness function parameters

We tested a wide range of values for our four fitness parameters ω, β, κ, and μ (Fig. 7) to identify the optimal set for describing the biological IINs. We systematically set each parameter set to zero, to completely remove that selection pressure from the fitness function, with results summarized in Fig. S3. All four were needed. As discussed, κ and μ were the most important and the most tightly coupled for capturing the detailed local structure of the IINs (Fig. 4, Fig. S3). We initially varied μ over 0.2–2 and κ from 1–10, later decreasing the optimal search range to values of μ from 0.2–0.6, and κ from 1–3. Results were least sensitive to β, which we varied from 1 to 6, although past values of 2 there was not a significant change in resulting triangle motifs. β was least important because for sparse networks such as the IINs, triangle motifs are already relatively rare to sample. Results were sensitive to ω, which was varied over 0.001 to 1. However, the dependence on ω was straightforward to interpret, as it only controlled the number of edges and was largely uncoupled from other network features.

### Quantifying network degree distributions

We generated the spectrum of networks ranging from homogenous to scale-free using a single parameter (α) via the method of Goh *et al*.^64^. We term this parameter α the “preferential attachment exponent” (PAE) of the network. A PAE = 0 corresponds to a Poisson (random) network with λ = 〈k〉, and PAE = 1 roughly corresponds to a power-law (scale-free) network with γ = 2. We reverse fit the degree distribution of our sampled IINs by generating networks with specific P.A.E.s for comparison. Degree distributions for 11 values of the P.A.E. (0, 0.1, 0.2 … 1) were generated by building 30 networks (per P.A.E.) with the same number of nodes and edges as the IIN. Least χ^2^ distance was used to choose the best-fit P.A.E. for the degree distribution of the given IIN. We had to modify the algorithm of Goh *et al*.^64^ to generate networks that did not contain orphan nodes, and this procedure is detailed in the Supplementary Text S3.

### Statistic for identifying ‘date’ vs ‘party’ hubs

The distribution of interfaces for a protein is calculated by normalizing the Stirling numbers of the second kind (see Supplementary Text S1 for definitions). We use this probability distribution to generate a statistic for identifying proteins with an unusually high (party hubs) or an unusually low (date hubs) number of interfaces. For a protein with degree *k* and U interfaces, we can calculate a *p*-value using a two-tailed test, given by

5 $$p \mbox{-} {\rm{value}}=Pr(t\le \frac{k+1}{2}-|{\rm{U}}-\frac{(k+1)}{2}|)+\Pr ({\rm{t}}\ge \frac{k+1}{2}+|{\rm{U}}-\frac{(k+1)}{2}|)$$

where *t* can take only integer values [1:k]. If *U* = (*k* + 1)/2, *p*-value $\equiv 1$.

In Table S1 we report these *p-*values per protein, indicating which proteins have an unusually small or large number of interfaces.

### Generation of alternate PPIN structures

Five variations of the CME network^20^ were used to test PPIN constraints on IIN sampling: a “dense” network with the same P.A.E. where 186 edges were added to the existing CME network, a “sparse” network also with a comparable P.A.E. where 93 edges were deleted, and a random version of each of the preceding three networks with the same number of proteins and PPIs using the Erdos-Renyi algorithm. Finally, a random version of the ErbB PPIN^4^ was also used.

### Phylogenetic analysis of yeast CME proteins and human ErbB proteins

To determine the evolutionary conservation of domains in the 56 yeast CME proteins and 127 human ErbB proteins, we collected orthologs of each protein, ran multiple sequence alignments with MAFFT^65^, and analyzed residue conservation with the ConSurf^66^ rate4site program (or web-server). To assign a conservation score to each domain, the average over all residues in the domain were taken (Table S3). Orthologs were constructed from BLAST^67^ searches against the UniRef90 clustered sequence database with an E-value cutoff of 0.0001. This approach to use BLAST searches against UniRef90 to identify orthologs across all species is the same as used in other conservation calculation approaches^66, 68^. Consistent with these approaches^68^, we kept only sequences that were similar in length to the query sequence (25% longer or shorter) and shared sequence identity of 35%-95% before performing the multiple sequence alignment (MSA).

Hub interfaces were defined in two independent ways: firstly, as any interface with 5 or more interactions (results shown in Table 2). Secondly, we used the statistic defined in Eq 5 to identify proteins with an unusually low number of interfaces given their connectivity, implying the presence of hub interfaces. The statistics were almost identical, with 89% and 71% of hub and non-hubs, respectively, being more conserved than average.

### Network rewiring between yeast CME proteins and human CME proteins

We constructed the CME interaction network for human homologs of the yeast proteins using the review of Weinberg *et al*.^38^ as a guide to functional homologs in metazoans. Most human homologs were identified directly from this review^38^, and in a few cases we supplemented this with human orthologs identified from the EggNOG database^69^, which were confirmed by BLAST searches of the yeast proteins against exclusively human proteins. Nine yeast proteins lacked human homologs (as was previously documented^38^) and the remaining 45 yeast proteins were matched with 64 human homologs, as compiled in Table S4. Interactions between these 64 proteins were then extracted from BioGRID. We also added 9 interactions involving actin or the Arp2/3 complex and removed 11 involving the Arp2/3 complex to be consistent with the publications used to make the interface assignments in yeast^20^ that involved crystal structures of metazoan homologs.

The yeast CME network contained 18 PPIs that were mediated through multiple duplicate binding modes (Fig. S6A). These interactions were found to be slightly more conserved than single binding mode interactions, with 9 conserved interactions, 4 lost due to a lost homolog, and 5 lost despite both proteins retaining homologs and domains.

Code for network sampling and analysis is available from github https://github.com/mjohn218/network_sampling_MC.

## Electronic supplementary material

Supplementary Information

Table S1

Table S2

Table S3

Table S4

Table S5
