# Supplementary material for: Protein-protein binding selectivity and network topology constrain global and local properties of interface binding networks
Source: Sci Rep. 2017 Jul 17;7:5631. doi: 10.1038/s41598-017-05686-2 (PMC5514078; doi:10.1038/s41598-017-05686-2)
Supplement: Supplementary file 1 — Supplementary Information [file 41598_2017_5686_MOESM1_ESM.pdf]

Supplementary Information for
"Protein-protein binding selectivity and network topology constrains global and local properties of interface binding networks"

David O. Holland[b], Benjamin H. Shapiro[a], Pei Xue[a], and Margaret E. Johnson[a*]

Table of Contents

Supplementary Text S1: Modified sampling approaches
Supplementary Text S2: Calculated network properties
Supplementary Text S3: Quantifying degree distribution without orphan nodes
Supplementary Text S4: Theoretical properties of IINs constrained to PPINs
Supplementary Text S5: Description of methods for constructing IINs from PPINs.

SI Figures S1-S7

Supplementary Tables S6-S8

Supplementary Tables S1 through S5 are separate .xlsx files:

Supplementary Table S1: Yeast CME and Human ErbB Protein node properties
Supplementary Table S2: Yeast CME and Human ErbB Protein and Interface networks
Supplementary Table S3: Yeast CME and Human ErbB Interface Residue Conservation analysis
Supplementary Table S4: Human CME orthologs and their PPIN
Supplementary Table S5: Rewiring of PPINs between Yeast and Human CME PPINs

Supplementary Text

S1. Modified IIN sampling approaches

1a Self-loop isolation: Our sampling procedure did not initially apply any penalty to highly connected self-binding interfaces, resulting in their random distribution in sampled IINs (Fig. S1). By adding a simple penalty against high connectivity for self-binding interfaces, we could reproduce the accurate isolation of these nodes without affecting other network properties.

1b Unbiased shuffle We also sampled IINs for a given PPIN but kept the number of interfaces per protein fixed as in the PPIN. The only move was then to allow edges to move between these interfaces. This sampling produced similar results to the unbiased sampling of the full range of IINs (Table S6).

1c Modified fitness function We modified our fitness function penalty on the total interfaces to test whether allowing larger fluctuations in the number of interfaces per protein would improve the IIN selectivity for scale-free or random PPINs. For the modified fitness function, absolute number of interfaces was penalized instead of interfaces per protein, such that the μ term of the fitness equation was changed to $e^{\mu(N_{interfaces}-N_{proteins})}$ and μ was lowered to 0.032 to produce a realistic number of interfaces.

S2. Properties calculated of networks

2a. Global clustering coefficient: Given by

$$C_{global,3} = \frac{3N_{triangle}}{N_{open}+N_{triangle}} \qquad \text{Eq S1}$$

where N_{open} is the number of open triplets and $N_{triangle}$ the number of closed triplets.

2b. Four-node motifs, or tetramers: These were enumerated by finding all four-node subgraphs connected by at least one path, and determining which of six possible architectures each subgraph matched: chain, square, hub, flag, 5-edge, or 6-edge. A single node may belong to more than one subgraph, but a subgraph of four nodes may only be classified as one of the six motifs. The ratios of the amount of each motif to the total number of tetramers were used as a global statistic of the likelihood of each motif. We refer to this as the motif frequency. The three subgraphs with clustering (flags, 5-edge, and 6-edge subgraphs) were grouped into a single frequency due to their rarity.

2c. Fragmentation The fragmenting or modularity of the network was quantified using the size of the largest component in the network. To normalize, we also calculated the percentage of network interfaces contained in the largest component.

S3. Quantifying network degree distribution without orphan nodes

3a. Comparison network without orphans. To establish the degree distribution of an observed network, we needed to best match that network with networks of the same size but varying degree distributions. To generate the networks for comparison (with N nodes and M edges), we had to modify the algorithm of Goh et al. to prevent orphan nodes (k=0). To summarize, beginning with N nodes, each node is assigned an individual weight of $1^{-\alpha}$, $2^{-\alpha}$, $3^{-\alpha}$ … $N^{-\alpha}$. Edges are then added by selecting two nodes with probabilities equal to the normalized weights. Self-edges

were allowed and if an edge already existed then another pair of nodes would be selected. To prevent orphans, we performed this procedure with *M-R* edges, and used the remaining *R* edges to connect the orphans back into the network using the same probabilities as above. If there were too many orphans to reconnect, the network was discarded and the procedure rerun. The optimal value of *R* was defined through a recursive formula that was based on the expected number of orphans produced by the unmodified algorithm. Specifically, we found $R = \lim_{n\to\infty} a_n$ where $a_n = \sum_{i=1}^{N}\left(1 - i^{-P.A.E.}/\sum_{j=1}^{N} j^{-P.A.E.}\right)^{2(M-a_{n-1})}$ and a_0=0. Without orphans, the sparse networks in particular (<k>≈1) were more similar to one another regardless of the P.A.E., since each node was required to have at least one connection.

S4. Theoretical properties of IINs constrained to PPINs The expected number of interfaces for a protein of degree *k* can be calculated from the probability mass function of such a protein having *n* interfaces, where *n* varies from 1 to *k*. We find this distribution is captured by normalizing the Stirling numbers of the second kind:

$$S_k^{(n)} = \frac{1}{n!}\sum_{i=0}^{n}(-1)^i\binom{n}{i}(n-i)^k. \qquad \text{Eq. S2}$$

The normalization factor is the Bell number, introduced in the main text, that counts the total number of ways to partition the *k* edges into interfaces,
$B_k = \sum_{n=0}^{k} S_k^{(n)}$. The expected number of interfaces for a protein of degree k is

$$\langle n\rangle_k = \sum_{n=1}^{k} n S_k^{(n)}/B_k \qquad \text{Eq. S3}$$

and values for proteins in both manually curated PPINs are reported in Table S1. The expected number of interfaces per IIN is then the sum over all the proteins, and is 200 for the CME PPIN and 411 for the ErbB PPIN (when duplicate edges are included-see Table S1).

The distribution of IIN sizes (in number of interfaces) for a given PPIN is the convolution over all proteins of their Stirling distributions. Each Stirling distribution is narrower and left shifted compared to a Binomial distribution, and their convolution results in an explosion of possible networks centered around the expected interface size. Sparse and dense IINs are then extremely rare.
The total number of IINs for a PPIN is the product of its protein's Bell numbers.

$$Total\ IINs\ possible = \prod_{j=1}^{N_{pro}} B_{\deg(j)} \qquad \text{Eq. S4}$$

where deg(*j*) is the degree of protein *j*. Scale-free PPINs will have significantly more types of IINs possible relative to a random PPIN given the very large Bell numbers of their hub proteins.

To determine the effect of PPIN structure on the degree distribution of IINs, we resorted to computational approaches, and used the unbiased MC sampling ($k_BT=\infty$). Results in Fig. S4 show that most IINs have a random degree distribution, but it is more probable to produce a scale-free IIN from scale-free PPINs than random PPINs.

S5. Different construction methods for biological IINs

5a. ErbB network We analyzed two versions of the ErbB network, the original version, where every phosphosite is a separate interface, and a reduced version, where copies of interfaces on a protein with the same specificity for binding partners were represented as a single interface. This reduced the network from 387 interfaces to 303, and from 545 edges to 417, mostly due to

the large number of phosphosites per protein that often were all targeted by the same kinase domains. The results of the motif selectivities were the same for both networks, but with the smaller size and smaller number of duplicated edges, the reduced IIN was easier to sample.

5b Automatically constructed networks. The automatically constructed IINs (Fig. S2) were downloaded from the studies of Wang et al (1) on the human structural interaction network (hSIN), and from the study of Deeds et al (2), whose cytoplasmic yeast SIN network was originally constructed by Kim et al (3). In both cases domains were assigned to the PPINs using the crystal structures of bound proteins complexes in *i*PFam. Since most protein complexes have not been crystallized, if the proteins in the PPIN contained domains with homologs that interacted, these were assigned as the predicted domain-domain interactions. Either because *i*PFam contains a limited number of linear motif interactions, or linear motifs are not recorded as known domains for specific proteins by PFam, assignments of binding sites such as PRDs and phosphosites as partners were not captured. Also, interfaces were assigned by domain, but the two are not synonymous because protein domains can be large and contain multiple, distinct binding interfaces.

5c. Automatic construction of the CME IIN Using the S. cerevisiae database on the INstruct website (4), we used the hSIN method of Wang et al to reconstruct the Clathrin-mediated endocytosis IIN. As Fig. S2d shows, only 44 interactions are present, compared to 206 in the manually curated network. Nearly all of the predicted domain-domain interactions are incorrect, or they are assigned to proteins that are not actually observed to bind directly to one another after reading the cited literature. Manual curation, while tedious, allows one to use biochemical data that identifies binding sites, whether a crystal structure exists or not.

SI Figures

Figure S1.

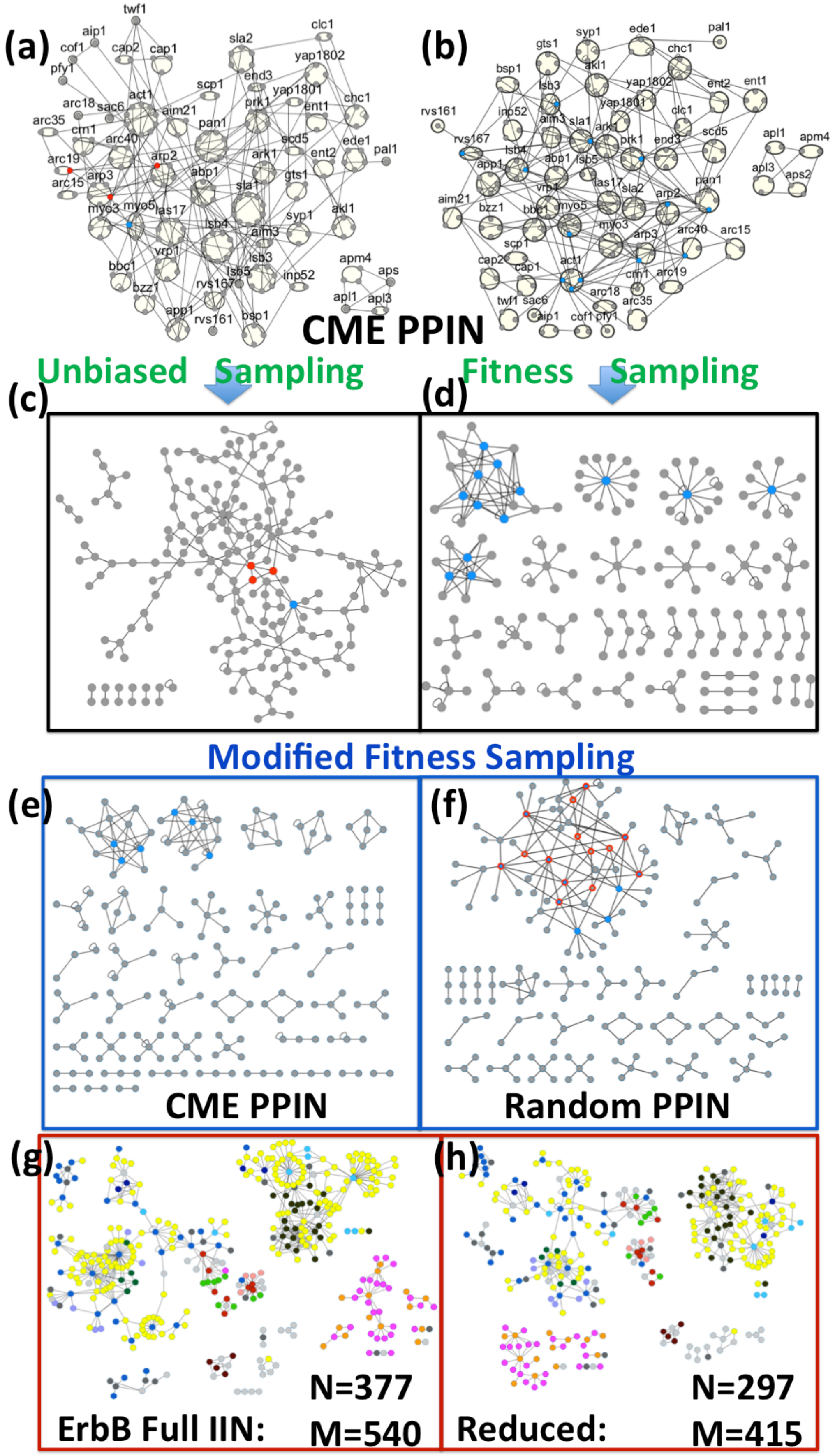

Figure S1. IIN structures from distinct sampling approaches have distinct structures.
The CME PPIN in both **a** and **b** is identical, but the number and distribution of interfaces on the proteins is different due to **(a)** unbiased sampling of interface networks and **(b)** fitness sampling of the interface networks. **c, d.** The interface interaction networks (IINs) of A and B are shown separated from the protein network. Unbiased (random) sampling of interface networks in **(c)** and fitness sampled result shown in **(d)**. Unbiased sampling shares no features in common with the biological IINs of Fig. 1, but with fitness sampling we can reproduce nearly all the properties of the biological IINs. **(e)** Modified fitness sampling produced similar results for the CME PPIN. **(f)** However, on the random PPIN, modified fitness sampling limits on total interfaces resulted in a significant number of triangle motifs (red nodes). **g,h** The ErbB IIN with domains colored as in main text has similar properties whether repeated interfaces are kept separate **(g)** or grouped **(h).** Hub nodes (k>7) are colored blue. Network figures were all prepared with Cytoscape (5), and site graphs required the AutoAnnotate App. Interactive files are available from our website: https://hollandnetworkmotif.wordpress.com along with associated data files.

Figure S2.

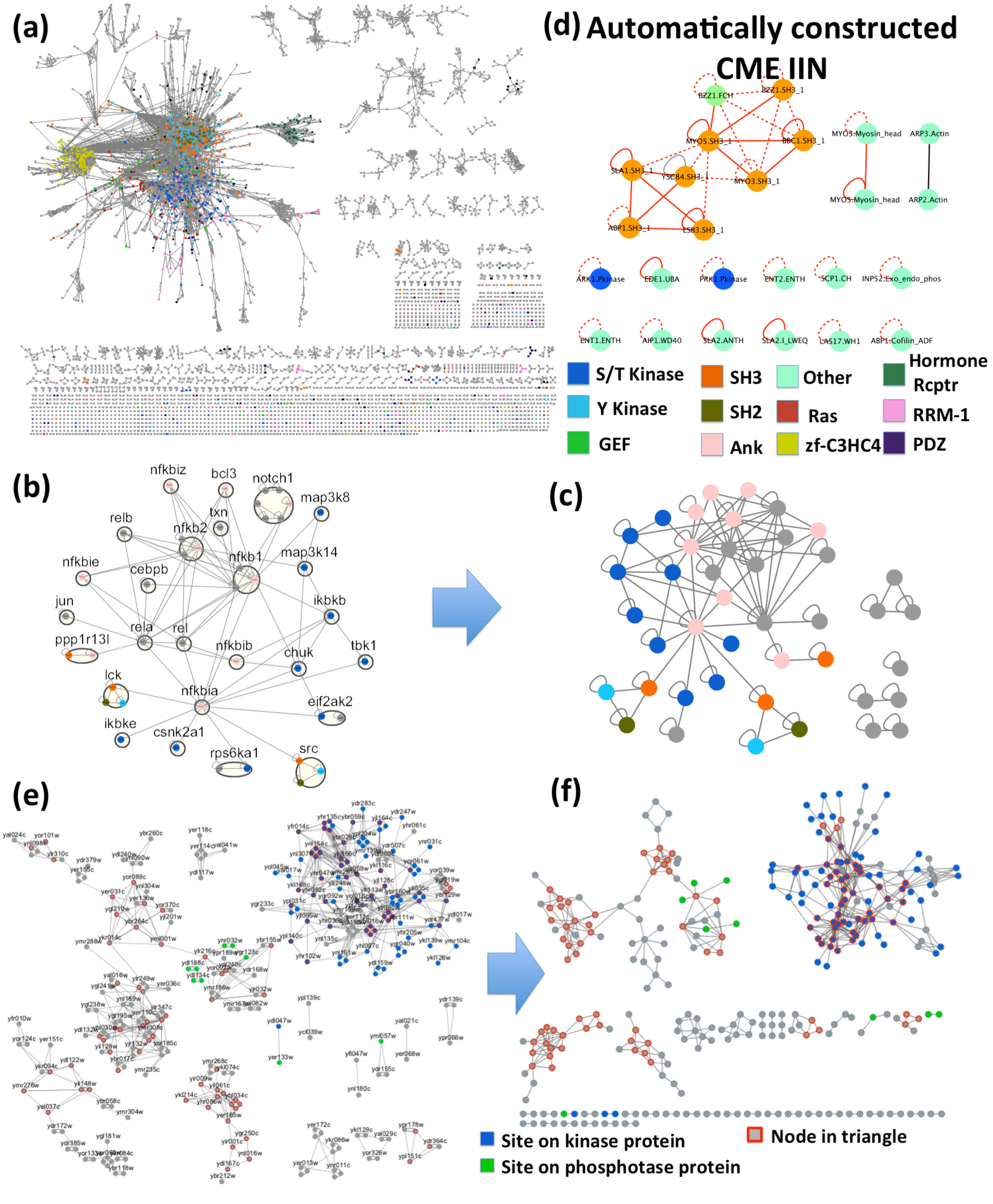

Figure S2. Automatically constructed IINs differ from manually curated networks. (a) The IIN from the human structural interaction network (1). Although it is fragmented, it is only so at a level equivalent to the protein network (Table 1). Hence the IIN does not create many distinct interface modules like the manually curated networks. (**b)** Site graph and isolated IIN **(c)** for a small portion of the human structural interaction network of (A). **(d)** Yeast CME network reconstructed with the *S. cerevisiae* INstruct database (4). The IIN is clearly much smaller than

the manually curated Yeast CME network of Fig. 1C, and many of the assigned interactions from the Instruct database do not occur in the manually curated network. Black edges indicate correct domain interactions. Red edges are for incorrect domain interactions, but correct PPIs. Dashed red edges indicate interactions we removed because they weren't in the reference literature or were found to not be direct. Gray edges are interactions for domains we didn't define. **(e, f)** Yeast structural interaction network (3) with only the cytoplasmic proteins (2). The IIN **(f)** is again only fragmented at the same level as the PPIN. Interface types are not annotated in the published data, so interfaces on kinase and phosphatase proteins are highlighted. There are no self-loops and many triangles.

Figure S3.

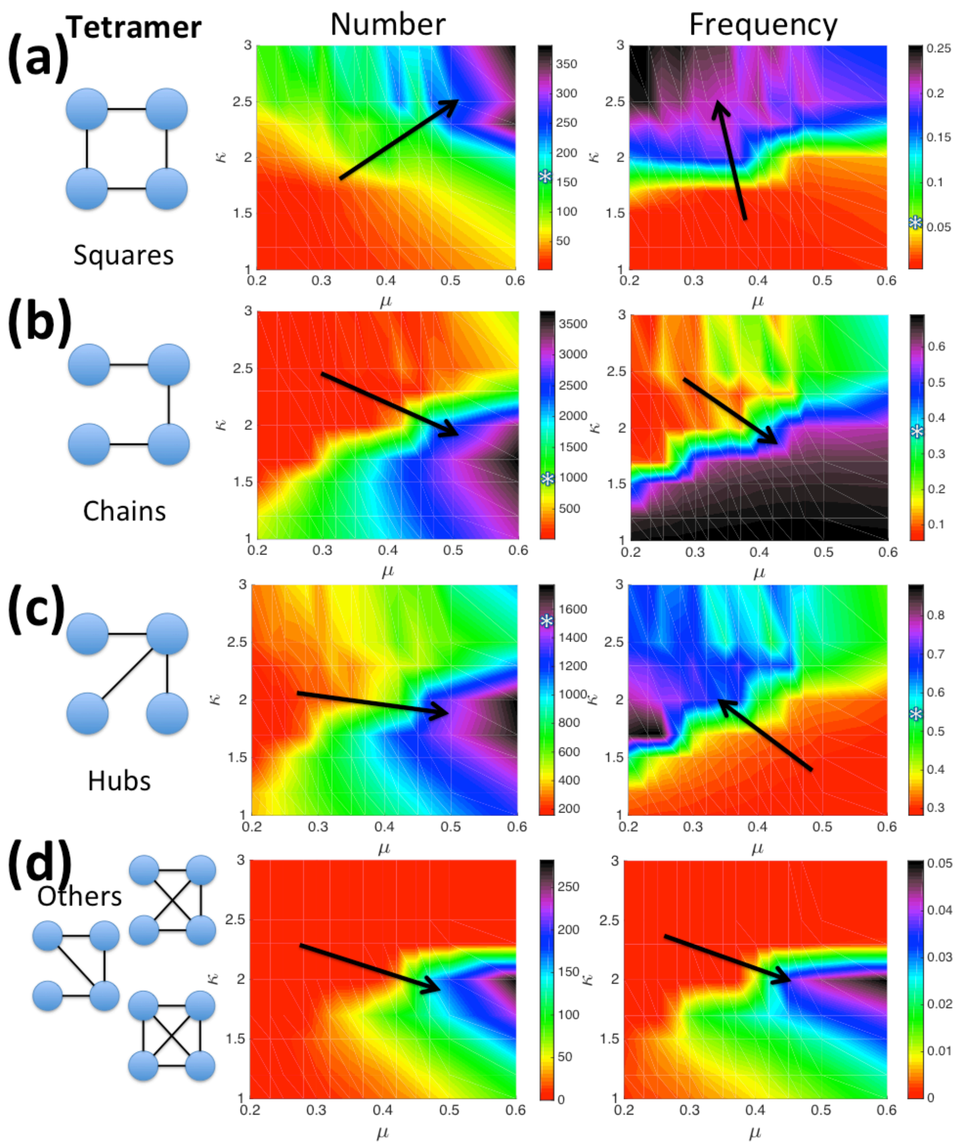

(e)

β	μ	κ	ω	Effects
ON	ON	ON	OFF	More edges, more interfaces (<k>≈2), more squares (edges close chains)
ON	ON	OFF	ON	Lower PAE (0.3-0.4), less squares and more chains, giant component with 80-90% of network
ON	OFF	ON	ON	Lower PAE (0.4-0.5), more interfaces, less squares. Fragmented network of pairs and small star hubs
OFF	ON	ON	ON	More clustering/triangles, especially when starting from dense extreme. PAE unaffected.
OFF	ON	OFF	ON	Moves towards dense extreme (one interface per protein)
ON	OFF	OFF	ON	No clustering, binomial network (PAE=0-0.2), <k>≈2 (network size constrained by strength of ω), few squares

Figure S3. Fitness function parameters determine number and frequency of four-node motifs in sampled IINs. The number and frequency of **(a)** square motifs **(b)** chain motifs **(c)** hub motifs and **(d)** the three remaining tetramer types, all from IINs sampled with a fitness function where the parameters κ and μ are varied. Arrows indicate direction of increase of motif

frequencies. Results from Monte Carlo sampling performed with $k_BT=1$ on the CME PPIN (Fig. 1a). The other two parameters not shown on axes were set to $\beta=4$ and $\omega=0.1$ for these simulations. The last three tetramer types in **(d)** ('Others') include clustering – penalized by β – which only occur as μ is increased since this drives the IIN closer to the PPIN in structure. The white stars indicate the statistics of the real CME IIN, which contains no clustering. **(e)** Each of the four parameters in the fitness function (κ, μ, β, ω) control structural aspects of the sampled IIN structures (Methods). By turning off each parameter, we illustrate how the networks respond with fewer biasing forces on their structural elements. Without any control of interfaces (μ is off), interfaces are more abundant and the network is relatively disconnected, whereas without any square bias (κ is off), squares are uncommon, chains are not penalized, and therefore the network does not fragment, resulting in a giant component.

Figure S4.

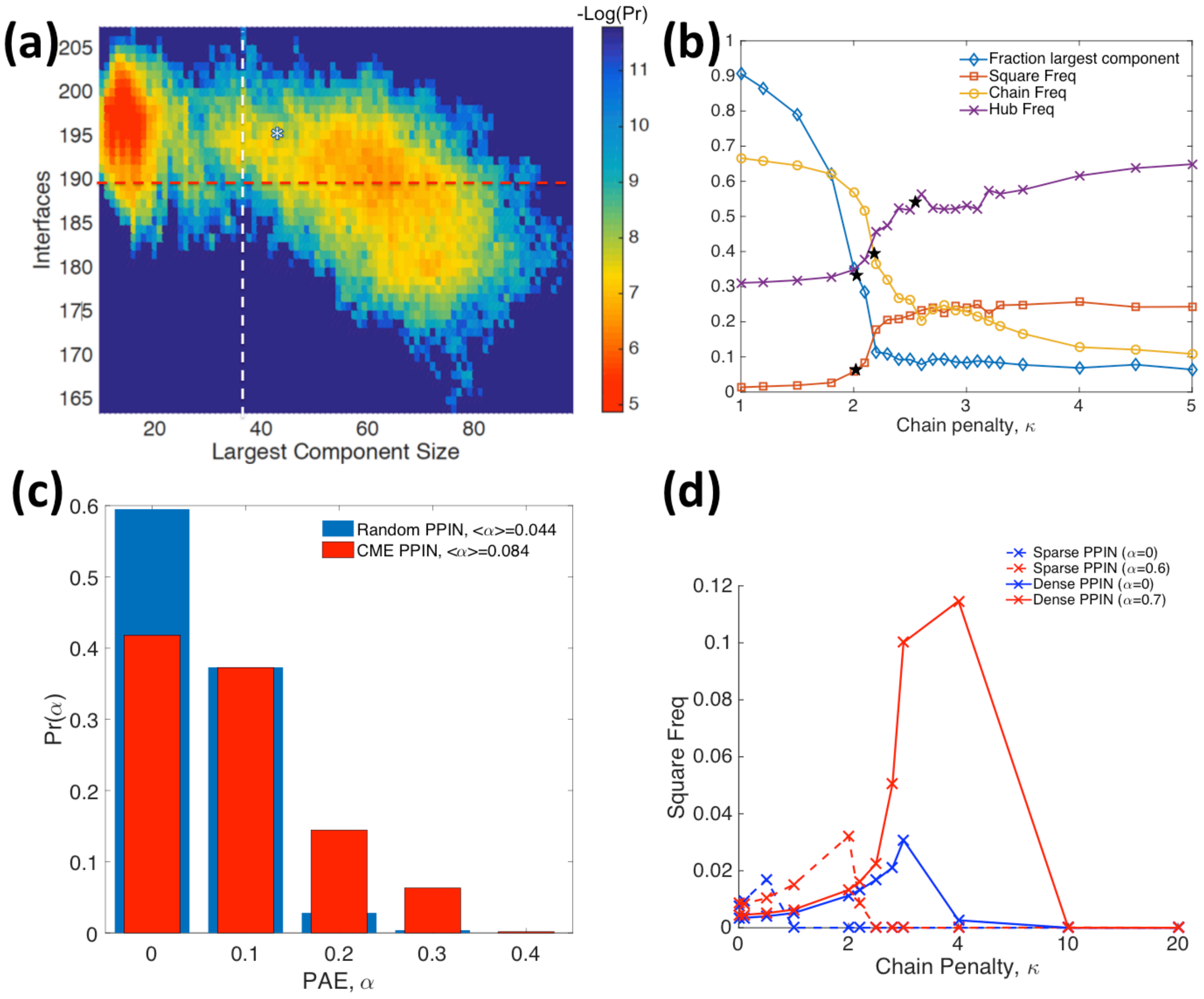

Figure S4. IIN properties vary as the structure of the PPIN varies.

(a) Probability distribution of IINs sampled with a fixed parameter set (the optimal parameters) as a function of the reaction coordinates of largest component size and interface number shows two basins. IINs were sampled for the CME PPIN, and the white star indicates the statistics of the actual CME IIN. The white line divides the networks 50-50. The sampled networks had to pass a threshold (red line) to transition left. **(b)** Effects of κ on fragmentation and tetramer frequency in IINs sampled from the ErbB PPIN. Black stars indicate observed values. Other parameters used were: β=4, μ=0.5, ω=0.025, k_BT=1. **(c)** The distribution of PAEs with unbiased sampling ($k_BT=\infty$) is broader for scale-free like (red bars) PPINs, meaning scale-free like IINs are more common. **(d)** For both sparse and dense PPINs, the scale-free like version (red curves) produced a higher square frequency over nearly all κ values. Other parameters were β=4, μ=0.45, ω=0.1, and k_BT=1.

Figure S5.

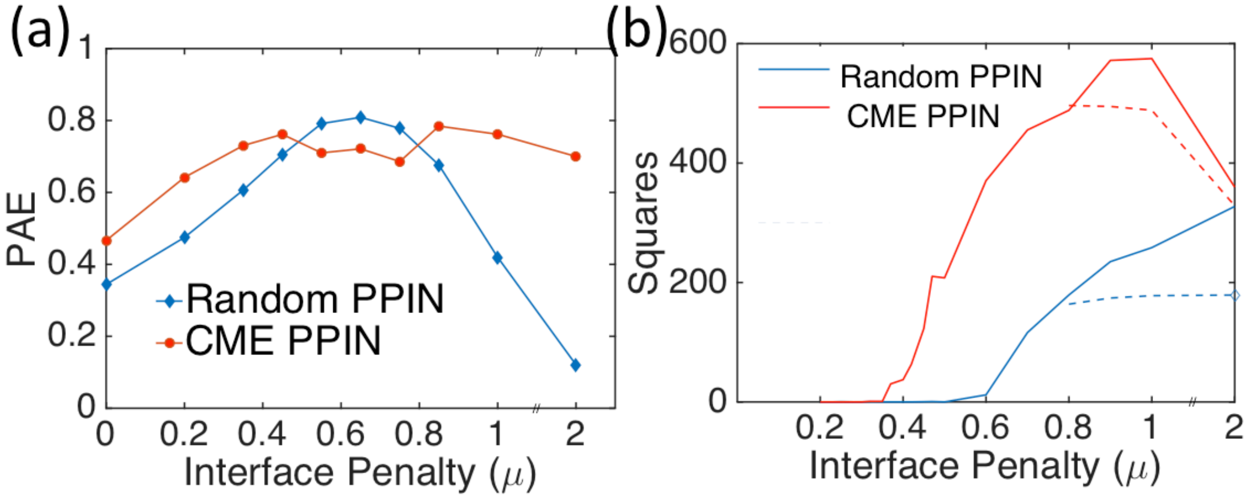

Fig S5. Random PPINs have more constraints in selecting fit IINs.
(a) By varying the parameters of the fitness function, we verified that random PPINs (blue curves) are more limited than scale-free like PPINs (red curves) for producing sampled IINs with large PAEs. Large PAEs indicate hub *interfaces* are present. **(b)** Random PPINs also limit the frequency of square motifs in their IINs. Squares appear readily in the scale-free like PPIN (red curve) thanks to the presence of hub proteins which produce more tetramers in the PPIN that can become squares in the IIN. Edge duplication is one mechanism to produce additional squares (solid lines vs no edge duplication in dashed lines), usually by closing chains into squares.

Figure S6.

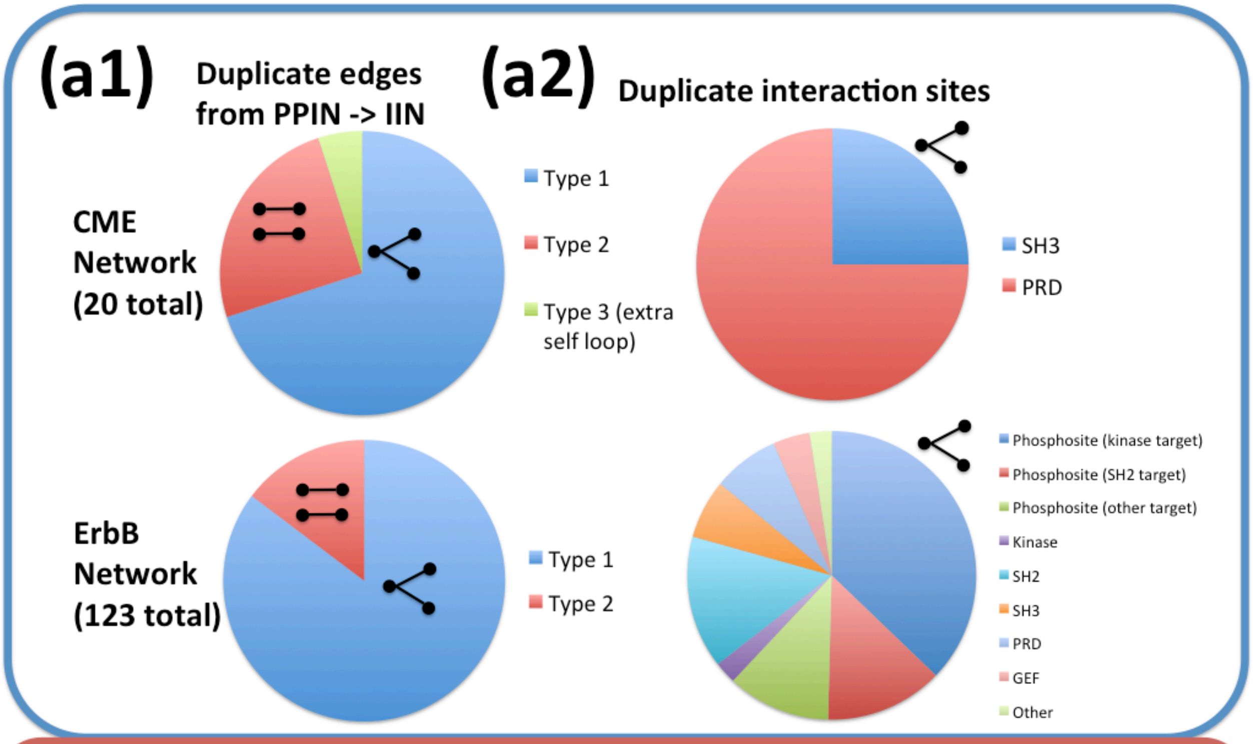

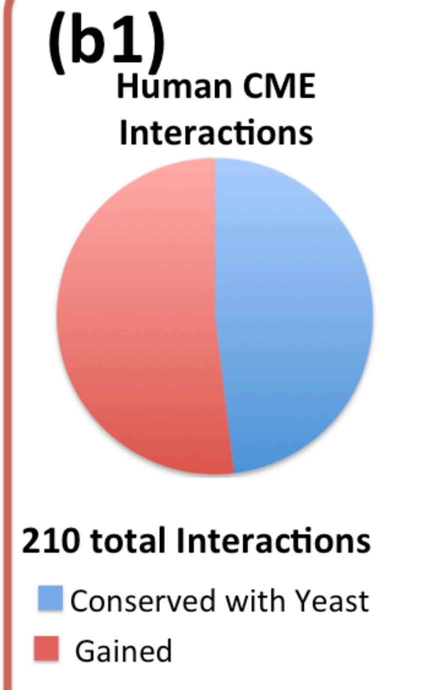

(b2)

	Human proteins	Gained inter-actions	% alignment with yeast	Sequence conser-vation
AP2 complex	AP2B1, AP2A2, AP2A1, AP2M1, AP2S1	38	AP2B1: 64%	32%
Kinases	BMP2K, AAK	9	All: ~30%	38%
SH3 containing proteins	DBNL, FNBP1L, PACSIN2, SH3YL1, MYO1E, MYO1F, BIN1, SH3GL2, SH3KBP1, ITSN1	14	-	-
PRD containing proteins	WAS, WASL, WIPF1, WIPF2, SH3KBP1, ITSN1	9	-	-
Actin Capping	CAPZA	20	94%	32%
Clathrin light chain	CLTA/B	18	~56%	19%
Synapto-janin	SYNJ1	7	50%	35%

Figure S6. Network rewiring from human to yeast CME networks is dominated by a few proteins and numerous PPIs are duplicated in the IINs due to repeated domain copies.
(a) Many PPIs in the CME and ErbB networks produce multiple edges in their IINs due mostly to repeated copies of the same domain type. **(a1)** These extra binding modes between protein pairs where the two edges share an interface (type I) outnumber modes involving separate interfaces for each edge (type II). **(a2)** Of the type I extra binding modes, about 75% result from multiple copies of unstructured binding sites (e.g. PRDs, phosphosites). **(b)** CME interactions of Human

functional homologs are compared to the Yeast interactome. **(b1)** About half the human interactions are conserved in yeast as well. **(b2)** Gained interactions were not most prominent in SH3 containing proteins, but were most heavily centered in the AP-2 complex. The AP-2 complex acquires a critical beta-appendage domain not present in yeast that acts as a hub interface in metazoans, binding multiple types of linear motifs (6) and clathrin. Both the actin capping protein and the clathrin light chains do not appear to make structural changes, but the low sequence conservation could drive acquisition of new partners to surface patches. Both lack canonically recognized binding domains.

Figure S7.

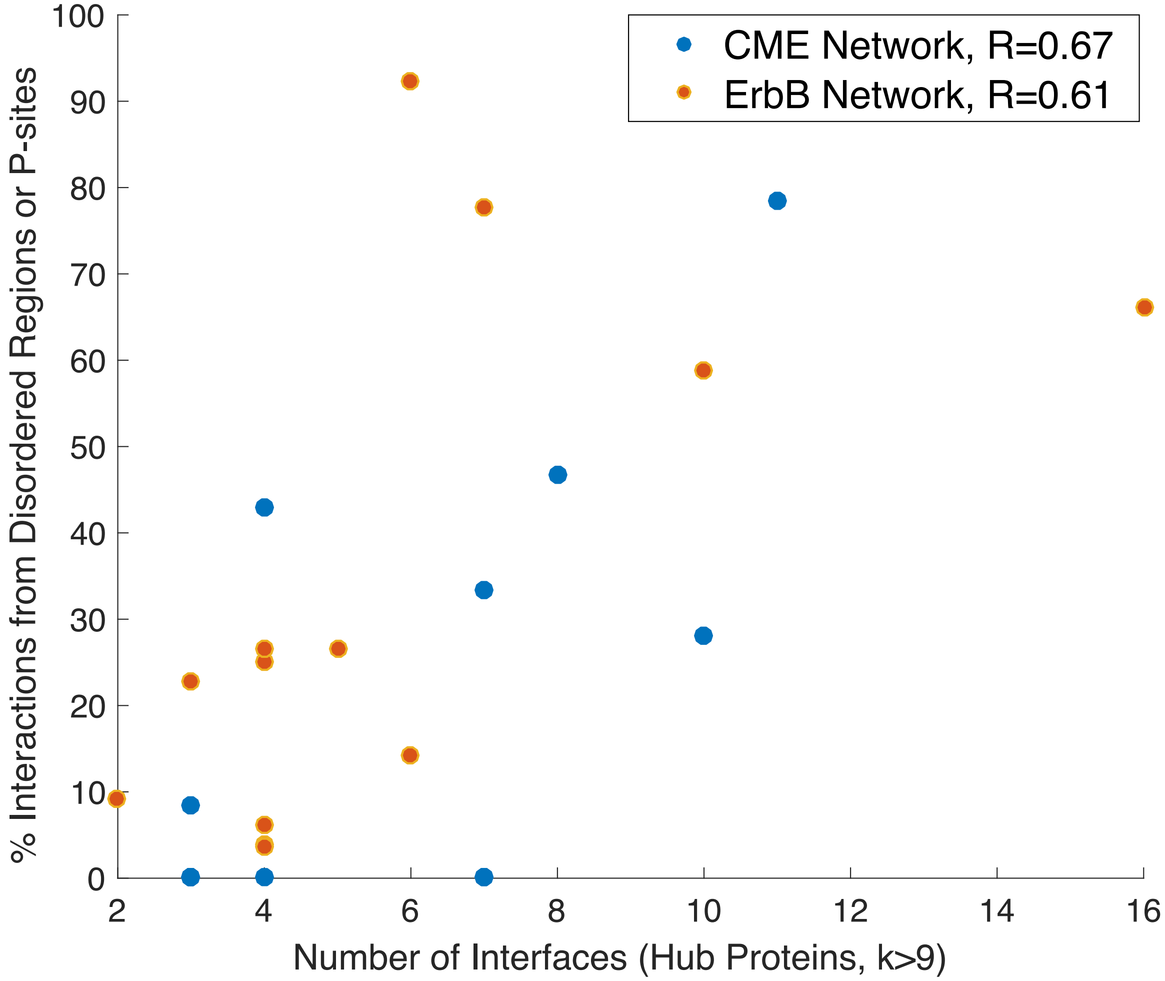

Figure S7. Hubs with many interfaces mediate interactions through disordered regions. Hub proteins in both the CME and ErbB networks lie on a spectrum between having multiple disordered interfaces, or having a small number of versatile structured interfaces (at least one usually a hub interface). As the plot shows, the more interfaces a hub has, the more likely it is that they mediate most of their interactions through disordered regions. This is in large part because hub proteins with more interfaces have more disordered interfaces (not shown). In contrast, hub proteins with few interfaces have a least one hub interfaces, which are highly conserved (Table S8) and thus rarely disordered. The % interactions from disordered regions consider only whether the hub protein itself used a disordered region. We note that for the ErbB network, we counted all phosphosites as disordered, although this may not be the case in reality. For the CME network, disordered regions included proline rich domains (PRDs), acidic domains, and clathrin boxes (7). For this particular analysis only, we considered larger hubs, so hub proteins had more than 9 binding partners. In our conservation analysis, we used two related but distinct definitions of hub proteins.

Table S6. Sampling with a fitness function reproduces properties of biological IINs.

	Yeast CME Protein Network				Human ErbB Signaling Network				
	Unbiased Shuffle[(a)]	Unbiased Sampling[(b)]	CME IIN	Fitness Sampling	Unbiased Shuffle[(a)]	Unbiased Sampling[(b)]	ErbB (Reduced) IIN	Fitness Sampling	ErbB Full IIN
Interfaces	195	193.7 ± 2.46 (Max 196)	195	192.1 ± 5.4	195	303.7±0.55 (Max 304)	297	308.1 ± 7.13	377
Edges	206	207.0 ± 0.17 (Max 207)	206	209.9 ± 2.8	206	419.8±0.38 (Max 420)	415	424.8 ± 13.1	540
Pref. Attach. Exp.	0.50 ± 0.043	0.086 ± 0.090	0.8	0.67 ± 0.08	0.49 ± 0.004	0.24 ± 0.07	0.7	0.81 ± 0.05	-
Largest Component (%)	75% ± 3.4%	82% ± 4.0%	23%	13% ± 10%	92% ± 2.4%	96% ± 2.0%	35%	13% ± 3.3%	38%
C_{Global}	0.015 ± 0.0099	0.016 ± 0.011	0	0.0048 ± 0.011	0.0044 ± 0.003	0.011 ± 0.0054	0.0015	0.0020 ± 0.0036	0.0014
Tetramers	1,306 ± 126	819.2 ± 91.9	2,743	1,139.7 ± 620	6,177 ± 273	4,312 ± 280.4	10,856	14,750 ± 3,254	38,626
Square	0.0033 ± 0.002	0.0021 ± 0.0016	0.061	0.16 ± 0.06	0.017 ± 0.020	0.0054 ± 0.0012	0.066	0.18 ± 0.026	0.03
Chain	0.67 ± 0.021	0.73 ± 0.021	0.37	0.30 ± 0.13	0.65 ± 0.01	0.72 ± 0.011	0.36	0.36 ± 0.044	0.16
Hub	0.32 ± 0.021	0.26 ± 0.020	0.56	0.53 ± 0.08	0.33 ± 0.0098	0.27 ± 0.010	0.57	0.45 ± 0.023	0.8
Other Tetramer	0.013 ± 0.009	0.0095 ± 0.0081	0.0	0.005 ± 0.01	0.0032 ± 0.0024	0.0094 ± 0.0050	6e-4	0.0019 ± 0.0037	5e-4

[a]Shuffling of edges while keeping the number of interfaces on each protein constant. No bias from a fitness function.

[b]Sampling without bias from a fitness function, i.e. random sampling of IINs for a given protein network.

Table S7. Statistics of best individual IINs for random vs scale-free like PPINs.

	Original Fitness Function		Modified Fitness Function	Original Fitness Function	
	CME PPIN	Random PPIN	Random PPIN	ErbB PPIN	Random (ErbB) PPIN
Interfaces	174	215	191	290	356
Edges	202	190	201	364	278
Pref. Attach. Exp.	0.8	0.7	0.7	1	1
Largest Component (%)	9.8%	5.1%	32%	11%	2.3%
C_{Global}	0	0	0.033	0	0
Tetramers	1534	463	2915	9423	234
Square	0.17	0.056	0.017	0.21	0.051
Chain	0.18	0.13	0.48	0.27	0.013
Hub	0.65	0.82	0.23	0.52	0.94
Other	0	0	0.28	0	0
Fitness Penalty (modified function)	74.62	357.9	251.5	---	---
Fitness Penalty (original function)	279.1	366.9	517.5	548.8	564.9

Table S8. Residue conservation analysis for Human ErbB and Yeast CME proteins

	More Conserved than Average	Score (0 is average, <0 is conserved)
**All CME and ErbB Domains/Interfaces**		
Hub Interfaces (55)	89%	-0.42±0.36
Non-hubs (371)	70%	-0.22±0.6
PRDs[a] (48)	41%	0.11±0.63
Residues not in domains (174 proteins)	15%	0.3±0.4
**Non-hub interfaces**		
Bind to hubs (212)	64%	-0.18±0.7
Do not bind hubs (159)	77%	-0.3±0.45

[a] Proline Rich Domains

SI References

1. Wang X, Wei X, Thijssen B, Das J, Lipkin SM, Yu H. Three-dimensional reconstruction of protein networks provides insight into human genetic disease. Nature biotechnology. 2012;30:159-64.
2. Deeds EJ, Krivine J, Feret J, Danos V, Fontana W. Combinatorial complexity and compositional drift in protein interaction networks. PLoS One. 2012;7(3):e32032.
3. Kim PM, Lu LJ, Xia Y, Gerstein MB. Relating three-dimensional structures to protein networks provides evolutionary insights. Science. 2006;314(5807):1938-41.
4. Meyer MJ, Das J, Wang XJ, Yu HY. INstruct: a database of high-quality 3D structurally resolved protein interactome networks. Bioinformatics. 2013;29(12):1577-9.
5. Smoot ME, Ono K, Ruscheinski J, Wang PL, Ideker T. Cytoscape 2.8: new features for data integration and network visualization. Bioinformatics. 2011;27(3):431-2.
6. Schmid EM, Ford MGJ, Burtey A, Praefcke GJK, Peak-Chew SY, Mills IG, et al. Role of the AP2 beta-appendage hub in recruiting partners for clathrin-coated vesicle assembly. Plos Biol. 2006;4(9):1532-48.
7. Dafforn TR, Smith CJ. Natively unfolded domains in endocytosis: hooks, lines and linkers. EMBO Rep. 2004;5(11):1046-52.
